# Thermodynamic Performance Analysis and Refrigerant Evaluation of Enhanced Cascade Vapor-Injection Refrigeration Systems

**DOI:** 10.3390/e28070747

**Published:** 2026-07-01

**Authors:** Jidong Li, Maolin Cai, Weiqing Xu, Guanwei Jia

**Affiliations:** 1 School of Automation Science and Electrical Engineering, Beihang University, Beijing 100191, China; ljdbuaa@buaa.edu.cn (J.L.);; 2 Pneumatic and Thermodynamic Energy Storage and Supply Beijing Key Laboratory, Beijing 100191, China; 3 School of Physics and Electronics, Henan University, Kaifeng 475004, China

**Keywords:** cascade refrigeration systems, energy efficiency, performance improvement, vapor injection, exergy analysis, alternative refrigerants

## Abstract

To enhance the compression efficiency and refrigerant flow capacity for low-temperature refrigeration applications, the vapor-injection strategy is innovatively synthesized with two-stage cascade refrigeration systems. Two cascade vapor-injection configurations with subcoolers and flash tanks (CSVIRS and CFVIRS) are compared with the conventional cascade refrigeration system (CCRS) through integrated thermodynamic simulations. The impacts of crucial temperature and injection parameters are comprehensively analyzed through energy and exergy methods, while the performance comparisons of various refrigerant combinations are also conducted. The coefficient of performance (*COP*) of the CFVIRS exceeds that of the CCRS and CSVIRS by 33.84% and 2.10% under the default condition. The cascade vapor-injection configurations exhibit a performance advantage at higher condensation temperature and lower evaporation temperature of the low-temperature cycle (LTC). The evaporation temperature of the high-temperature cycle (HTC) and injection pressures are examined with optimum solutions. Decreasing the entrainment ratio of the HTC and increasing the entrainment ratio of the LTC within appropriate ranges are beneficial for the refrigeration performance. R1270-R170 demonstrates superior energy and exergy performance, whereas R143a-R23 shows the highest improvement ratio among the compared refrigerants. The implementation of cascade vapor injection substantially reduces exergy destruction in the compression and expansion devices, while the exergy characteristics of various refrigerant pairs are extensively investigated.

## 1. Introduction

The refrigeration industry is pivotal in modern society, providing not only comfortable and healthy living environments but also playing a crucial role in food preservation and climate control [1]. In recent years, low-temperature refrigeration technologies have advanced significantly, driven by growing demands in sectors like cold chain logistics, biomedicine, natural gas liquefaction, etc. [2]. As a fundamental part of numerous industrial segments, the refrigeration sector currently accounts for approximately 17% of global electricity consumption [3]. According to the prediction from the International Energy Agency (IEA), this figure is expected to rise to 30% by 2050, indicating a substantial growth trajectory [4,5]. Attributed to the rapid growth of the world population and the ongoing advancement of industrialization, the strain on global power systems has intensified significantly [6]. Issues such as global warming, carbon dioxide emissions, ozone layer depletion, and chlorofluorocarbon-related pollution have become increasingly severe [7]. Refrigeration and air conditioning systems, which rely on external energy inputs to complete thermodynamic cycles, offer compelling energy efficiency advantages that can help optimize global energy consumption. Developing efficient, sustainable and environmentally friendly refrigeration systems is a pivotal challenge for advancing human society [8].

Recent years have witnessed growing research interest in ultra-low-temperature refrigeration applications [9]. Typically achieved through vapor compression refrigeration systems, this process involves cooling specific products or spaces to generally below −50 °C. In some extreme circumstances, it may even require temperature as low as −80 to −100 °C [10,11]. Vapor compression refrigeration systems are favored for their wide range of applications, ease of use, and cost-effectiveness [12]. However, when the refrigeration temperature drops below −40 °C, traditional single-stage vapor compression systems suffer from low evaporation pressure, high compression and throttling losses, deteriorated compressor operating conditions, and significantly reduced energy efficiency [13,14].

To reach lower refrigeration temperatures, it is typically necessary to employ auto-cascade or cascade vapor compression structures [15]. The auto-cascade method achieves staged refrigeration relying on the separation of mixed working fluids with different boiling points integrated in a single cycle [16]. Auto-cascade systems are relatively sophisticated and require advanced equipment, introducing significant challenges in charging and controlling different refrigerants precisely. As for cascade refrigeration systems, different refrigeration cycles utilize appropriate refrigerants to operate at individual pressure and temperature levels. Each stage employs an independent refrigeration compressor interconnected through the cascade heat exchanger, thereby attaining the desired temperature through a sequential cascading process [17]. The pressure ratio of each cycle is moderate, which contributes to the cooling performance and energy efficiency [18]. The structure and control principles are straightforward, ensuring that the system is well suited for various refrigeration applications in commercial and industrial domains. Conventional cascade refrigeration systems primarily employ a two-stage configuration. To reach lower temperatures, the cascade structure with three or more stages can be trialed, which increases the complexity of the structure and requires further verification of practicability [19,20]. The control of operational parameters is crucial for the energy efficiency of the system. In recent decades, researchers have conducted studies on various influencing factors of cascade systems [21,22]. Uusitalo et al. examined the performance of heat pumps with different refrigerant combinations, as well as the effects of the cascade heat exchanger temperature level, reaching the highest coefficient of performance (*COP*) of 3.08 by adapting R601 and R245fa in the high and low stage [23]. Kim et al. determined the optimal cascade temperature for the R134a-R410A cascade heat pump system using numerical and experimental methods, analyzing the effects of mass flow rate, pressure ratio, and heat exchange temperature difference [24]. Sun et al. conducted a comparative analysis of refrigerant selection for cascade refrigeration system, finding that the combination of R404A-R41 has higher *COP* and exergy efficiency than R404A-R23 [25]. Gholamian et al. proposed an NH_3_-CO_2_ cascade refrigeration system, noting that the CO_2_ expansion valve, compressor, and cascade heat exchanger are the key components for efficiency improvement, and the total exergy efficiency can be enhanced by 23.81% [26]. Sun et al. also conducted a comparative study on the performance of a three-stage cascade refrigeration cycle using various refrigerant combinations, including R717-R41-R1150, R152a-R41-R1150, R717-R170-R1150, R152a-R170-R1150, and R161-R170-R1150. The results indicated that R717, R152a and R161 exhibit superior performance in the high-temperature cycle, R41 and R170 can substitute for R23 in the medium-temperature cycle, and R1150 can effectively replace R14 in the low-temperature cycle [27]. Chen et al. developed a cascade system consisting of multi-stage coupled absorption chillers (ABCs) and adsorption chillers (ADCs) and conducted relevant experimental studies, achieving the maximum *COP* of 0.66 with the refrigeration capacity ratio of ABC to ADC set as 2.1 [28]. Deymi-Dashtebayaz et al. compared the performance of low global warming potential (GWP) refrigerant combinations of R161-R41, R1234yf-R41, R1234ze-R41, R161-R744, R1234yf-R744, and R1234ze-R744 in two-stage cascade systems, carrying out optimization analyses of *COP*, efficiency, and cost through the Pareto front curve [29]. Faruque et al. conducted an exhaustive comparative analysis of low-GWP hydrocarbon refrigerant collocations for cascade refrigeration systems, revealing a remarkable efficiency enhancement of at least 7.21% over findings in the existing literature by utilizing toluene for the high-temperature cycle and trans-2-butene for the low-temperature cycle [30]. Zhang et al. researched the application of an R134a-CO_2_ cascade heat pump system for cold climates, noting that the *COP* for producing 50 °C hot water at ambient temperatures of −5 °C and −45 °C can reach 3.07 and 1.60, respectively [31]. Chen et al. analyzed the feasibility of using environmentally friendly refrigerant combinations of R717-R170, R717-R41, and R717-R1150 as alternatives to R404A-R23 in two-stage cascade refrigeration systems, proving that R717-R170 demonstrates the optimal performance, with *COP* enhanced by 18.58% [32]. Ganesan et al. analyzed a two-stage cascade heat pump system utilizing novel natural azeotropic refrigerant pairs of R600-R744 and R601-R744, reaching a *COP* and optimization ratio of 4.5 and 36% [33]. Yilmaz et al. analyzed the improving effect of the low-GWP refrigerant combination of R1233ZDE-R290 in cascade applications enhanced with nanoparticles, including carbon nanotubes (CNT), copper oxide (CuO), and titanium dioxide (TiO_2_), finding that CuO can provide the most significant improvement [34]. Ji et al. discussed the selection of environmentally friendly refrigerants for low-temperature refrigeration applications at −80 °C, including R290-R170, R290-R1150, R717-R170, R717-R1150, R1270-R170, R1234yf-R170, and R1234yf-R1150, verifying that the R290-R170 exhibits the best efficiency performance [35]. Hosseinnia et al. conducted a comparative analysis of refrigerants for high-temperature cascade heat pumps. With R718 utilized in the high-temperature stage, six low-GWP refrigerants (R1234ze(Z), R1233zd(E), R1336mzz(Z), R600, R600a, and R601) were evaluated in the low-temperature cycle, and the R718-R600 combination represents the best energy performance [36].

In order to further enhance the system’s efficiency and compressor stability under extreme conditions of low evaporation temperature and high pressure ratio, an innovative solution of vapor-injection technology has been promoted in recent years [37]. Differing from the conventional refrigeration cycles, the vapor-injection refrigeration system incorporates an injection pathway with intermediate temperature and pressure via the economizer/subcooler or flash tank [38]. The process initiates with the compressor inhaling refrigerant from the evaporator and undergoing primary compression. Subsequently, the compressed output merges with the injected flow to fulfill an intermediate cooling procedure before entering the second period of compression [39]. This method effectively alleviates the compressor’s problems of limited suction flow and discharge overheating. By implementing a stepped-compression strategy, the compressor’s efficiency and the energy performance of refrigeration system can be feasibly enhanced; this process has received gradually increasing attention and has been increasingly researched [40,41]. d’Angelo et al. carried out a theoretical evaluation of a vapor-injection refrigeration system using a non-azeotropic mixed refrigerant of R290/R600a, showing that the *COP* increased by an increment of 16–32% compared to the basic cycle with various mixture concentrations [42]. Zheng et al. also conducted an improvement study on the vapor-injection cycle with a cascade condenser using various non-azeotropic mixtures, reporting that the R290/R600a (50%/50%) mixture can improve the *COP* by 1.9% and 2.6% compared to the flash tank cycle and the hybrid cycle [43]. Carvalho et al. compared the performance of various refrigerant mixtures, including R170, R290, R600 and R600a, in a flash tank vapor-injection refrigeration cycle, pointing out that the mixture of R600/R290 (60%/40%) is the most favorable choice, with an optimal *COP* of 4.8 [44]. Zou et al. analyzed the optimal intermediate temperature and injecting branch temperature of the vapor-injection system using R245fa-R410A, verifying that the *COP* of the system ranges from 1.16 to 1.58 when heating to 140 °C with ambient temperature ranging from −10 to 20 °C [45]. Tang et al. conducted experiments of vapor-injection improvements for linear compressors, noting that under the condition of evaporation and condensation temperature at −20 °C and 50 °C, the system’s *COP* can reach 2.06, with a 28% improvement over the traditional cycle [46]. Ning et al. conducted experimental tests on a vapor- and two-phase injection heat pump with a rotary compressor, finding that within the evaporation temperature range of −30 to 0 °C, the heating capacity and *COP* can be improved by 23.76–42.80% and 4.22–8.96% compared to the traditional cycle [47]. Zeng et al. carried out simulations and experimental evaluations of the rolling piston compressor with enhanced vapor injection for heat pump systems, finding that when the evaporation temperature is between −20 and −25 °C, the heating capacity and *COP* can be improved by 26–28.6% and 5.65–6.1% [48]. Maeng et al. conducted a comparative efficiency analysis of the vapor-injection system using R134a, R152a and R1234yf, pointing out that R152a demonstrates superior energy efficiency and environmental performance [49]. Wang et al. compared the vapor-injection mode with the vapor-return mode for an inverter air-source heat pump and validated the advantages of the vapor-injection method, which can improve the heating capacity and *COP* by 6.6% and 3.4% [50].

Given the continuous demands of lower refrigeration temperatures, the increases in operating temperature range and pressure ratio pose severe challenges to the compressor’s performance. Vapor-injection technology is competent in reducing the energy loss caused by excessive compression ratio and inadequate flow rate, thereby enhancing the stability and efficiency of the system. Despite extensive studies on cascade systems and vapor-injection technology separately, their combined applications lack systematic investigations, while the optimizations of injection configurations and refrigerant selections are also requisite for performance improvement. This paper focuses on improving two-stage cascade refrigeration systems through vapor-injection modifications, establishing a comparative analysis of subcooler and flash tank injection methods. The performance evaluations of different refrigeration systems and various refrigerant selections are discussed in detail, based on the factors of condensation temperature, evaporation temperature, injection pressure, and entrainment ratio. Through thermodynamic simulations, the optimization potential for the energy and exergy efficiency can be determined, thereby providing a theoretical basis for further experimentation and practical application of novel refrigeration systems.

## 2. Cycle Descriptions

**Figure 1:**
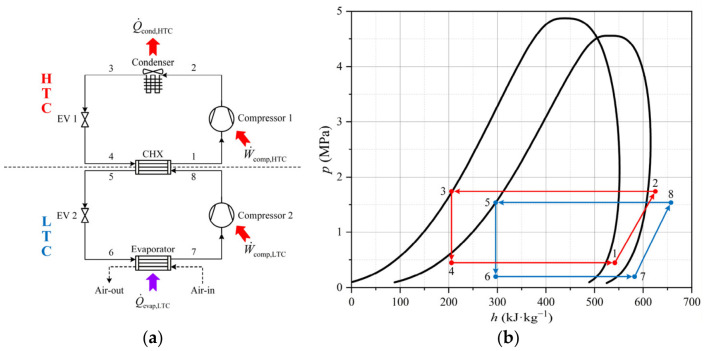
(**a**) The schematic diagram of CCRS; (**b**) The *p*-*h* diagram of CCRS.

This paper initially analyzes the conventional cascade refrigeration system (CCRS) as shown in Figure 1a, with the schematic *p*-*h* diagram illustrated in Figure 1b. The basic components of the first refrigeration stage include a compressor, a condenser, an expansion valve, and a cascade heat exchanger (CHX) [51]. After being compressed to a superheated high-pressure status (state 2), the refrigerant gets condensed to saturated liquid (state 3) and passes through expansion valve 1 to accomplish the isenthalpic expansion procedure (state 4). The CHX serves as the evaporator of the high-temperature cycle (HTC) and the condenser of the low-temperature cycle (LTC), enabling heat transfer across the stages to balance the condensation heat of the LTC. The refrigerant finally gets inhaled into compressor 1 to get compressed (state 1). The configuration of the LTC is similar to that of the HTC, and the evaporator delivers the final refrigeration output.

**Figure 2:**
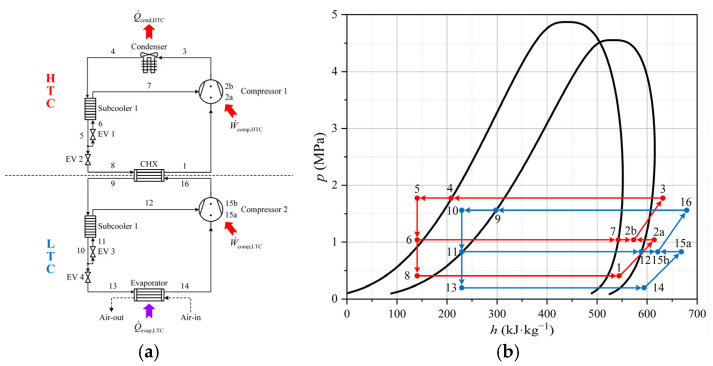
(**a**) The schematic diagram of CSVIRS; (**b**) The *p*-*h* diagram of CSVIRS.

**Figure 3:**
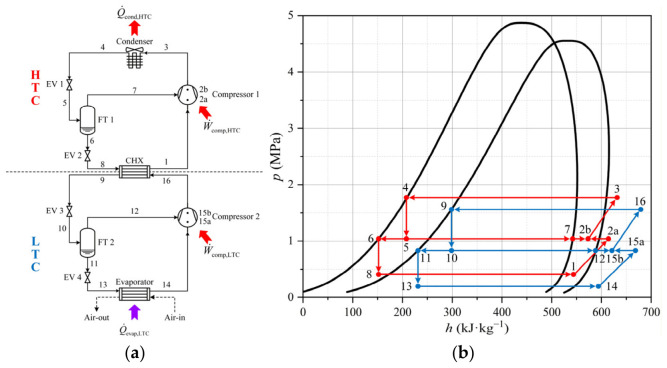
(**a**) The schematic diagram of CFVIRS; (**b**) The *p*-*h* diagram of CFVIRS.

Originating from the CCRS, the improvement primarily requires replacing conventional compressors with vapor-injection compressors. The main strategies consist of the subcooler method and flash tank method. The specific structures of the cascade subcooler vapor-injection refrigeration system (CSVIRS) and cascade flash tank vapor-injection refrigeration system (CFVIRS) and their schematic *p*-*h* diagrams are shown in Figure 2 and Figure 3, respectively. The operating principles are described as follows:

(1) Cascade subcooler vapor-injection refrigeration system (CSVIRS): In the HTC, after getting compressed to a superheated high-pressure status (state 3), the refrigerant gets condensed to saturated liquid (state 4). Then, the refrigerant passes through the subcooler 1 and divides into two streams (state 5). One stream gets throttled by expansion valve 1 to state 6 in order to absorb heat in subcooler 1 to state 7, which is subsequently injected into compressor 1 via the intermediate suction port. The other stream gets throttled by expansion valve 2 to state 8. The refrigerant, now in a cold two-phase condition, flows into the CHX to provide refrigeration for the condensation process of the LTC. The output stream is then drawn back into compressor 1 (state 1), where it undergoes the first compression stage to a mixed pressure state (state 2a), blends with the injected flow under constant pressure (state 2b), and then finishes the second compression process before entering the condenser. The operating principle of the LTC is similar to that of the HTC.

(2) Cascade flash tank vapor-injection refrigeration system (CFVIRS): Similar to a CSVIRS, the refrigerant gets compressed and condensed to saturated liquid (state 4). After getting throttled by expansion valve 1 to state 5, the two-phase stream enters flash tank 1 (FT 1) and gets separated into liquid flow (state 6) and gas flow (state 7). The gas flow is injected into compressor 1 via the intermediate suction port, and the liquid flow gets further throttled by expansion valve 2 to a cold two-phase status (state 8). After the evaporation process in the CHX, the output stream gets drawn back into compressor 1 (state 1), which undergoes similar injection and two-step compression procedures like the CSVIRS. The structure of the LTC is also identical to that of the HTC.

Implementing the vapor-injection strategy enhances the performance of vapor compression refrigeration by effectively achieving two-stage compression within a single-stage cycle. This modification reduces the compression ratio, thereby improving thermodynamic efficiency and lowering the discharge temperature. The CSVIRS and CFVIRS methods rely on subcoolers or flash tanks to establish vapor-injection bypasses. The former uses subcoolers to neutralize the residual sensible heat after condensation, thus enhancing the evaporation capacity. The latter employs gas–liquid separation to reduce the enthalpy prior to the main expansion and recycles the intermediate gas component for injection. Based on the aforementioned cascade models, comprehensive investigations into the factors influencing vapor-injection enhancement are required to support further optimizations and discussions.

## 3. Mathematical Models

### 3.1. Assumptions

Based on thermodynamic analysis methodologies, this paper establishes energy and exergy models for the aforementioned cycles, with appropriate simplifications. The specific assumptions mainly include [52,53,54]:

(1) The flows are regarded as one-dimensional and steady-state.
(2) The throttling processes in expansion valves are assumed to be ideal isenthalpic.
(3) The outlet state of the condenser is assumed to be that of a saturated liquid, and the superheat of the evaporator outlet is fixed as 5 °C.
(4) The outlet states of flash tanks are assumed to be saturated gas/liquid.
(5) The extra pressure and heat losses are negligible.
(6) The compression processes in the compressors are adiabatic and non-isentropic, with the isentropic efficiency *η*_comp_ used to estimate the irreversibility.
(7) The reference state is set as *T*_0_ = 25 °C, and *p*_0_ = 101.325 kPa.
(8) The changes in the kinetic and potential energy can be ignored.
(9) The temperature difference between the evaporation temperature and the cooled air (∆*T*_evap_), and the temperature difference between the evaporation temperature of HTC and the condensation temperature of LTC (∆*T*_CHX_), are all fixed as 5 °C.
(10) The temperature differences at the cold end of subcoolers (∆*T*_sub_) are set to 5 °C.
(11) All the heat exchangers work under the counterflow mode.

### 3.2. Energy Model

Based on the steady state assumption, the mass and energy balance equations of each component can be represented as follows:

(1) $$\sum {\dot{m}}_{\mathrm{in}}=\sum {\dot{m}}_{\mathrm{out}}$$

(2) $$\sum {\dot{m}}_{\mathrm{in}}(h_{\mathrm{in}}+\frac{u_{\mathrm{in}}^{2}}{2\times 1000})+{\dot{Q}}_{\mathrm{in}}+{\dot{W}}_{\mathrm{in}}=\sum {\dot{m}}_{\mathrm{out}}(h_{\mathrm{out}}+\frac{u_{\mathrm{out}}^{2}}{2\times 1000})+{\dot{W}}_{\mathrm{out}}+{\dot{Q}}_{\mathrm{out}}$$

Neglecting the potential and kinetic energy, the energy equation can be simplified as:

(3) $$\sum {\dot{m}}_{\mathrm{in}}h_{\mathrm{in}}+{\dot{Q}}_{\mathrm{net}}=\sum {\dot{m}}_{\mathrm{out}}h_{\mathrm{out}}+{\dot{W}}_{\mathrm{net}}$$

where ${\dot{Q}}_{\mathrm{net}}$ and ${\dot{W}}_{\mathrm{net}}$ represent the net heat absorption and net output work. The concept of entrainment ratio is defined as the ratio of the mass flow rate of the injection bypass ${\dot{m}}_{\mathrm{inj}}$ to the main stream ${\dot{m}}_{\mathrm{main}}$, and the entrainment ratios of HTC and LTC are defined as:

(4) $$\mu_{\mathrm{HTC}}=\frac{{\dot{m}}_{7}}{{\dot{m}}_{3}}=\frac{{\dot{m}}_{7}}{{\dot{m}}_{1}+{\dot{m}}_{7}}$$

(5) $$\mu_{\mathrm{LTC}}=\frac{{\dot{m}}_{12}}{{\dot{m}}_{16}}=\frac{{\dot{m}}_{12}}{{\dot{m}}_{12}+{\dot{m}}_{14}}$$

The default injection pressure for each cycle is presumed to be the geometric mean of the evaporation and condensation pressure in accordance with the relevant literature, which requires further evaluation [55]:

(6) $$p_{\mathrm{inj}}=\sqrt{p_{\mathrm{evap}}\cdot p_{\mathrm{cond}}}$$

Based on the aforementioned assumptions and the first law of thermodynamics, the energy conservation equations of each component can be summarized, respectively, as follows [56]:

- Compressor:

The compression work for each cycle of the system can be expressed as:

(7) $${\dot{W}}_{\mathrm{comp}}={\dot{m}}_{\mathrm{comp}}(h_{\mathrm{comp},\mathrm{out}}-h_{\mathrm{comp},\mathrm{in}})={\dot{m}}_{\mathrm{comp}}(h_{\mathrm{comp},\mathrm{out},\mathrm{is}}-h_{\mathrm{comp},\mathrm{in}})/\eta_{\mathrm{comp}}$$

The isentropic efficiency of the compressor can be determined referring to [57]:

(8) $$\eta_{\mathrm{comp}}=0.874-0.0135r_{\mathrm{comp}}$$

where *r*_comp_ refers to the compression ratio of the compressor. In the simulation model, the vapor-injection compression process for each stage is implemented by two compressors in series with an intermediate isobaric mixer, which diverts vapor from the flash tank or subcooler.

- Condenser:

The condenser is specified as an air-cooled type, and the heat transfer rate is calculated as:

(9) $${\dot{Q}}_{\mathrm{cond}}={\dot{m}}_{\mathrm{cond}}(h_{\mathrm{cond},\mathrm{in}}-h_{\mathrm{cond},\mathrm{out}})$$

- Subcooler:

According to the heat transfer relation and ignoring the heat leak to the environment, the heat balance of subcoolers can be obtained:

(10) $${\dot{m}}_{\mathrm{sub},\mathrm{cold}}(h_{\mathrm{sub},\mathrm{cold},\mathrm{out}}-h_{\mathrm{sub},\mathrm{cold},\mathrm{in}})={\dot{m}}_{\mathrm{sub},\mathrm{hot}}(h_{\mathrm{sub},\mathrm{hot},\mathrm{in}}-h_{\mathrm{sub},\mathrm{hot},\mathrm{out}})$$

- Cascade heat exchanger (CHX):

The cascade heat exchanger provides a bridge for the refrigeration output of the HTC to balance with the condensation heat of the LTC, and the energy equation is similar to that of the subcooler:

(11) $${\dot{m}}_{\mathrm{CHX},\mathrm{cold}}(h_{\mathrm{CHX},\mathrm{cold},\mathrm{out}}-h_{\mathrm{CHX},\mathrm{cold},\mathrm{in}})={\dot{m}}_{\mathrm{CHX},\mathrm{hot}}(h_{\mathrm{CHX},\mathrm{hot},\mathrm{in}}-h_{\mathrm{CHX},\mathrm{hot},\mathrm{out}})$$

- Flash tank (FT):

As for a CFVIRS, according to the energy balance of gas–liquid phase separation, the energy equation of the flash tank can be expressed as:

(12) $${\dot{m}}_{\mathrm{FT},\mathrm{in}}h_{\mathrm{FT},\mathrm{in}}={\dot{m}}_{\mathrm{FT},\mathrm{gas},\mathrm{out}}h_{\mathrm{FT},\mathrm{gas},\mathrm{out}}+{\dot{m}}_{\mathrm{FT},\mathrm{liq},\mathrm{out}}h_{\mathrm{FT},\mathrm{liq},\mathrm{out}}$$

The entrainment ratio of the CFVIRS can be determined by the input vapor quality of the flash tank based on the following assumption (4):

(13) $$\mu_{\mathrm{CFVIRS}}=x_{\mathrm{FT},\mathrm{in}}=\frac{{\dot{m}}_{\mathrm{FT},\mathrm{gas},\mathrm{out}}}{{\dot{m}}_{\mathrm{FT},\mathrm{in}}}=\frac{{\dot{m}}_{\mathrm{FT},\mathrm{gas},\mathrm{out}}}{{\dot{m}}_{\mathrm{FT},\mathrm{gas},\mathrm{out}}+{\dot{m}}_{\mathrm{FT},\mathrm{liq},\mathrm{out}}}$$

- Expansion valve (EV):

Referring to the assumption of isenthalpic expansion, the energy equation of the expansion valve is:

(14) $$h_{\mathrm{EV},\mathrm{in}}=h_{\mathrm{EV},\mathrm{out}}$$

- Evaporator:

The evaporation capacity of the evaporator can be defined as:

(15) $${\dot{Q}}_{\mathrm{evap}}={\dot{m}}_{\mathrm{evap}}(h_{\mathrm{evap},\mathrm{out}}-h_{\mathrm{evap},\mathrm{in}})$$

The *COP* of the aforementioned systems can be expressed as (subscripts corresponding with Figure 2 and Figure 3, respectively):

(16) $$COP_{\mathrm{CCRS}}=\frac{{\dot{Q}}_{\mathrm{evap}}}{\sum {\dot{W}}_{\mathrm{comp}}}=\frac{{\dot{m}}_{6}(h_{7}-h_{6})}{{\dot{m}}_{1}(h_{2}-h_{1})+{\dot{m}}_{7}(h_{8}-h_{7})}$$

(17) $$COP_{\mathrm{CSVIRS}/\mathrm{CFVIRS}}=\frac{{\dot{Q}}_{\mathrm{evap}}}{\sum {\dot{W}}_{\mathrm{comp}}}=\frac{{\dot{m}}_{13}(h_{14}-h_{13})}{\left[{\dot{m}}_{1}(h_{2a}-h_{1})+({\dot{m}}_{1}+{\dot{m}}_{7})(h_{3}-h_{2b})]+[{\dot{m}}_{14}(h_{15a}-h_{14})+({\dot{m}}_{12}+{\dot{m}}_{14})(h_{16}-h_{15b})\right]}$$

The improvement ratio of *COP* is defined as:

(18) $$\varepsilon_{COP}=\frac{COP_{\mathrm{CSVIRS}/\mathrm{CFVIRS}}-COP_{\mathrm{CCRS}}}{COP_{\mathrm{CCRS}}}\times 100\%$$

### 3.3. Exergy Model

Exergy is defined as the maximum theoretical work produced by a cycle when it comes to equilibrium with the reference condition of the environment. Exergy analysis serves as a critical procedure which identifies the key sources of exergy destruction and assesses the performance limits of the system to propose efficiency enhancement strategies [58]. Based on the second law of thermodynamics, the exergy model can be simplified as:

(19) $$\dot{E}x=\dot{m}[(h-h_{0})-T_{0}(s-s_{0})]$$

$T_{0}$, $h_{0}$, and $s_{0}$ represent the temperature, specific enthalpy and entropy values at the reference state. The equilibrium equation for each component can be established to evaluate the destruction rate [59,60]:

(20) $$\dot{E}x_{\mathrm{de}}=\sum \dot{E}x_{\mathrm{in}}+{\dot{Q}}_{\mathrm{net}}(1-\frac{T_{0}}{T})-{\dot{W}}_{\mathrm{net}}-\sum \dot{E}x_{\mathrm{out}}$$

The exergy equations of different components are listed in Table 1. The exergy destruction percentage is defined as the ratio of the exergy destruction in a particular component to the total exergy input of the system:

(21) $$\delta_{\mathrm{ex},\mathrm{de}}=\frac{\dot{E}x_{\mathrm{de}}}{\sum {\dot{W}}_{\mathrm{comp}}}\times 100\%$$

The exergy output of the system is contributed by the refrigeration procedure of the evaporator, and the temperature difference ∆*T*_evap_ should be considered to measure the heat transfer loss. The total exergy efficiency can be expressed as [61]:

(22) $$\eta_{\mathrm{ex}}=1-\frac{\sum \dot{E}x_{\mathrm{de}}}{\sum {\dot{W}}_{\mathrm{comp}}}=\frac{{\dot{Q}}_{\mathrm{evap}}\left|1-\frac{T_{0}}{T_{\mathrm{evap}}+\Delta T_{\mathrm{evap}}}\right|}{\sum {\dot{W}}_{\mathrm{comp}}}$$

### 3.4. Refrigerant Selections

Considering the key performance parameters mentioned in previous papers, numerous factors must be investigated when selecting refrigerants. As for low-temperature refrigeration systems, it is essential to ensure the critical metrics such as the thermodynamic and transport properties, energy performance, environmental impacts, safety, stability, and cost within feasible scopes. According to the Kigali Amendment to the Montreal Protocol, hydrofluorocarbon consumption and production must be phased down to 20% of the baseline level by the 2040s. This mandatory reduction drives a global transition toward eco-friendly refrigerants with zero ODP and low GWP, fundamentally reshaping the traditional refrigerant market [62,63]. To reach the target refrigeration temperature while balancing the compression ratio of each stage, the normal boiling points (NBPs) of the HTC and LTC are referred to as being around −45 and −85 °C. Based on the physical, chemical, and ecological assessment and comparison, the prospective refrigerants are presented in Table 2. All the selected refrigerants are harmless to the ozonosphere. Chosen as the representatives of HC natural refrigerants of the two stages, R1270 and R170 demonstrate advantages in terms of lower GWP and higher latent heat capacity, but their combustibility imposes inevitable limitations on application safety, which also restricts the promotion of R41. Although R143a, R32, and R23 exhibit relatively higher GWP values, their safety profiles and operational advantages in low-temperature freezing systems and industrial refrigeration applications remain irreplaceable. Further modifications can be implemented by utilizing non-azeotropic or azeotropic refrigerants to adjust thermodynamic performance more flexibly, but the cost of computational complexity and working instability should be seriously evaluated. Comparative discussions of the performance of cascade vapor-injection refrigeration systems with different refrigerant combinations will be conducted based on the aforementioned energy equations.

### 3.5. Simulation Model Settings

**Figure 4:**
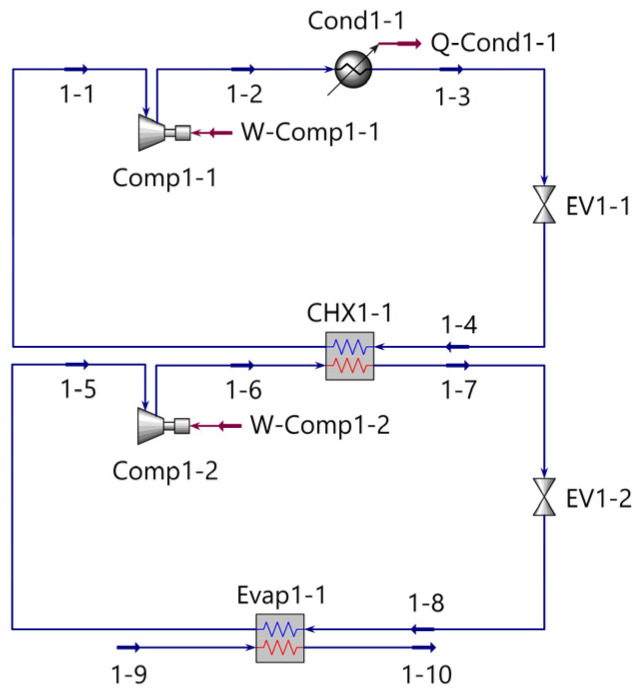
The basic simulation flow diagram of CCRS.

**Figure 5:**
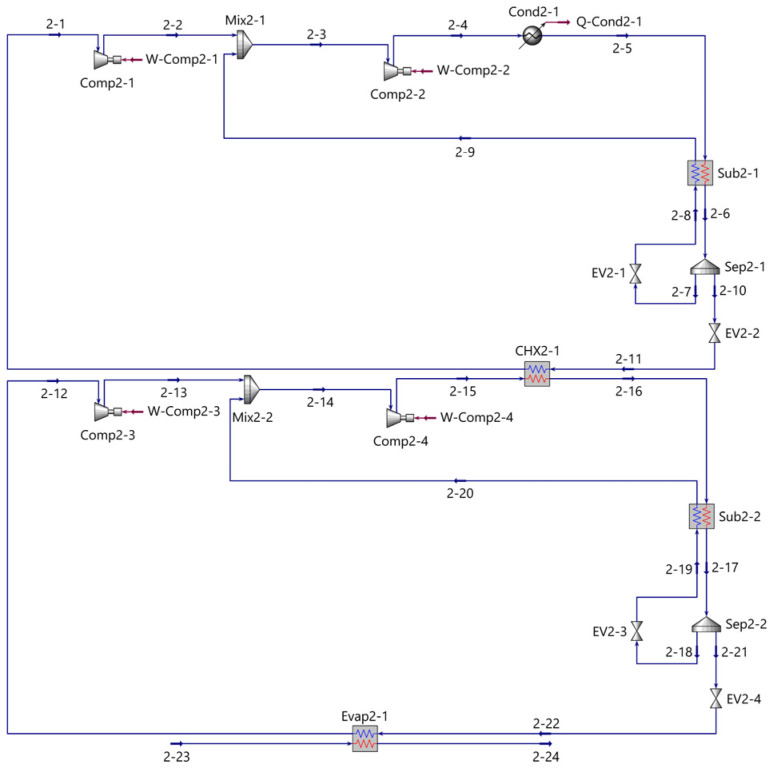
The basic simulation flow diagram of CSVIRS.

**Figure 6:**
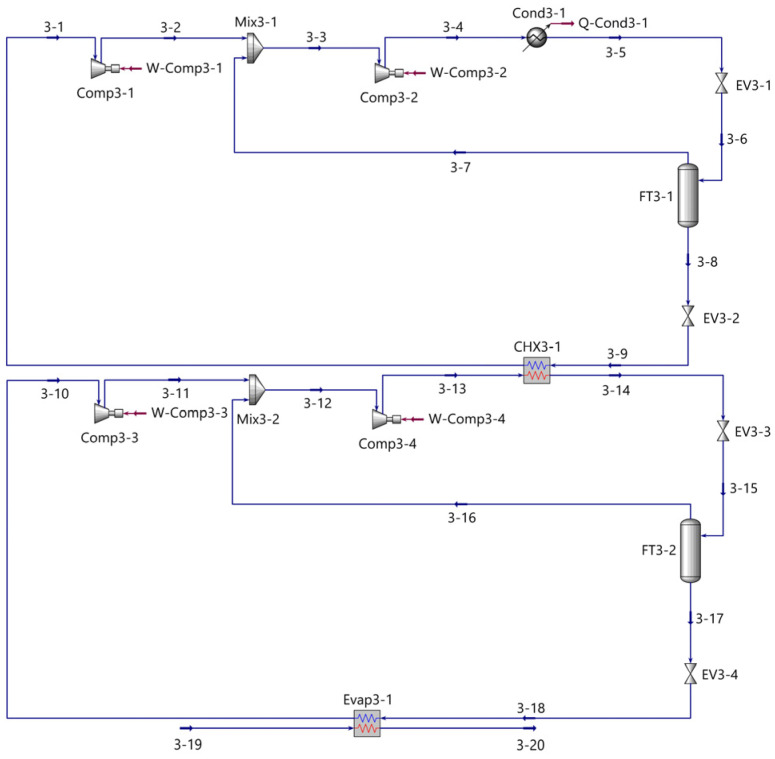
The basic simulation flow diagram of CFVIRS.

**Figure 7:**
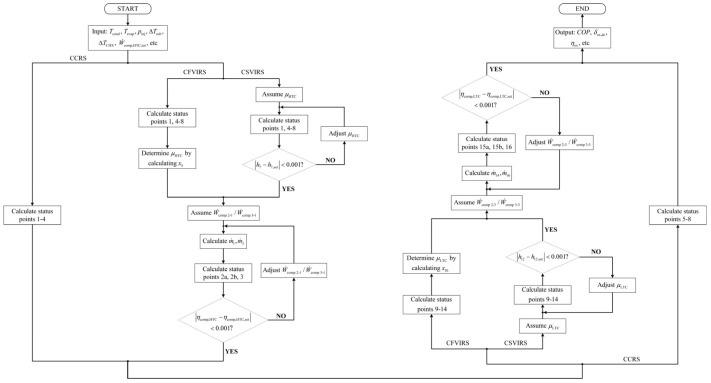
Calculation flow charts of different cascade refrigeration systems.

The cascade vapor-injection system’s performance is primarily affected by parameters such as the condensation temperature (*T*_cond_), the evaporation temperature (*T*_evap_), the injection pressure (*p*_inj_), and the entrainment ratio (*μ*_inj_). All the simulation models of the CCRS, CSVIRS, and CFVIRS are well established in Aspen HYSYS V15.0 software, and the basic flow charts of these systems are displayed in Figure 4, Figure 5 and Figure 6. REFPROP property package (Version 10.0), a thermophysical property database developed by the National Institute of Standards and Technology (NIST), is embedded through the Aspen Physical Property System to ensure calculation reliability [64]. The power input of the HTC is determined to be 3 ps (2.2 kW) for all systems. The typical and default working condition is selected as *T*_cond_ = 30 °C, *T*_evap,HTC_ = −40 °C, and *T*_evap,LTC_ = −80 °C, with *p*_inj_ initialized as Equation (6). For the subcoolers in the CSVIRS, the outlet states of the cold side are initialized as saturated vapor. The typical refrigerant pair of R143a-R23 is initially selected to evaluate the refrigeration characteristics. Moreover, the injection inlets of compressors of the CSVIRS are initialized as saturated gas, but the phase composition is variable while analyzing the effect of entrainment ratio. The calculation of the CCRS derives from the aforementioned basic energy balance equations, featuring a relatively simple procedure. Meanwhile, the computational processes of the CSVIRS and CFVIRS are relatively complex, with additional iterative adjustments of flow and power allocations to approximate the actual operating conditions of the cascade vapor-injection cycles. The corresponding calculation flow charts of different systems are shown in Figure 7.

To verify the accuracy and reliability of the simulation models, the operating parameters and design conditions from reference [65], which analyzed the performance of a CCRS system using various refrigerant pairs, are replicated using the model shown in Figure 4. The *T*_evap,LTC_, ∆*T*_CHX_, and ${\dot{Q}}_{\mathrm{evap}}$ are set to −60 °C, 5 °C, and 10 kW, respectively, with R1270–R170 selected as the example refrigerant pair. As *T*_cond_ rises from 20 to 40 °C, the resulting *COP* values are compared, and the percentage of relative error is listed in Table 3. The relative errors remain consistently between −1.16% and −0.98%, which sufficiently attests to the logical correctness and computational reliability of the proposed models.

## 4. Results and Discussions

### 4.1. Effect of Condensation Temperature T_cond_

**Figure 8:**
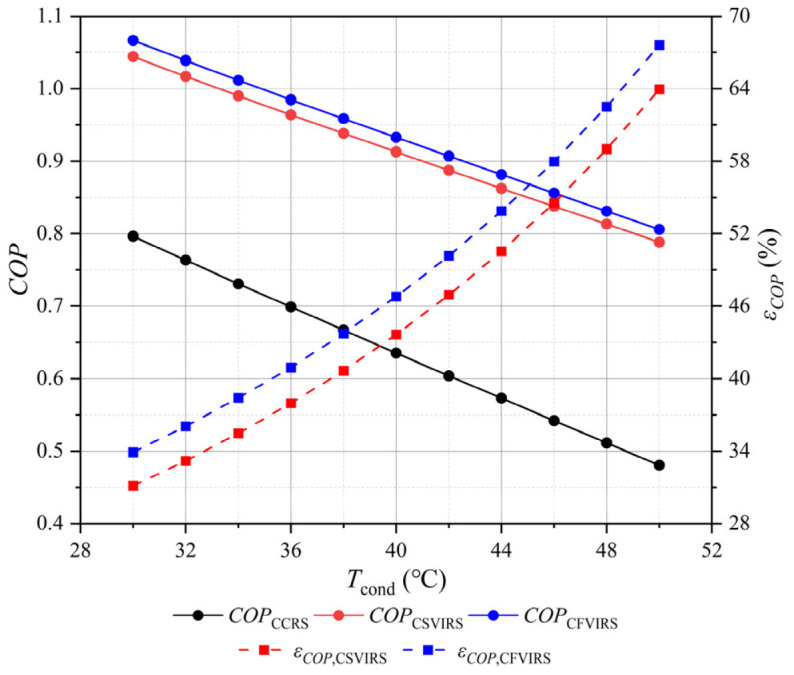
The effects of *T*_cond_ on *COP* and *ε_COP_*.

The condenser plays an essential role for the refrigeration system to discharge the compression heat to the environment or thermal storage medium. The operating condition of the condenser is decisively related to the compression ratio and efficiency. A comparative study is conducted about the impact of *T*_cond_ on the refrigeration performance. *T*_cond_ varies from 30 to 50 °C, with *T*_evap,HTC_ and *T*_evap,LTC_ maintained at −40 °C and −80 °C. The curves of *COP* and *ε_COP_* within the given range of *T*_cond_ are indicated in Figure 8. As *T*_cond_ increases, the *COP* of each system suffers a noticeable decline due to the rise in compression ratio and deterioration of compression efficiency, and the CSVIRS and CFVIRS are advantageous, with higher *COP* compared with the CCRS. As for *T*_cond_ = 30 °C, *COP*_CFVIRS_ reaches 1.066 while the *COP*_CSVIRS_ and *COP*_CCRS_ are 1.044 and 0.796, and the superior percentages of *COP*_CFVIRS_ are 2.13% and 33.92%. Furthermore, the *ε_COP_* of the CSVIRS and CFVIRS become more sufficient at higher *T*_cond_, which indicates the superiority of vapor-injection method under extreme operating conditions.

**Figure 9:**
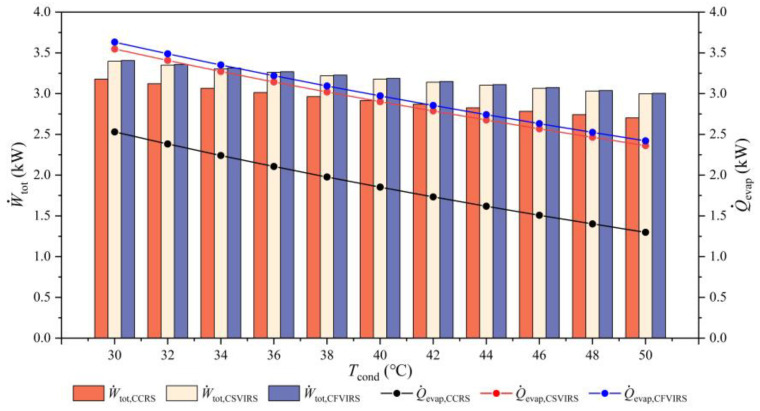
The effects of *T*_cond_ on ${\dot{W}}_{\mathrm{tot}}$ and ${\dot{Q}}_{\mathrm{evap}}$.

In order to analyze the energy characteristics of different systems, the variations in total compression work ${\dot{W}}_{\mathrm{tot}}$ and refrigeration output ${\dot{Q}}_{\mathrm{evap}}$ are also illustrated in Figure 9. Based on the fixed power assumption of the HTC, the rises in condensation temperature and pressure result in more specific compression power consumption and exacerbate compression efficiency, which significantly reduce the main flow and refrigeration output of the HTC. Accordingly, the main flow of the LTC also gets suppressed, attributed to the reduction in heat transfer in the CHX, and the final evaporation capacity suffers a notable deterioration. Within the discussed range of *T*_cond_, the ${\dot{W}}_{\mathrm{tot}}$ of the CFVIRS varies from 3.00 to 3.41 kW, which is 0.22–0.28% and 7.19–11.17% higher than the CSVIRS and CCRS. The ${\dot{Q}}_{\mathrm{evap}}$ of the CFVIRS ranges from 2.42 to 3.63 kW, which surpasses the CSVIRS and CCRS by 2.41–2.45% and 43.55–86.33%. The critical deficiency of ${\dot{Q}}_{\mathrm{evap}}$ intensifies the inferiority of the CCRS. Furthermore, the downtrend of ${\dot{Q}}_{\mathrm{evap}}$ is rather more prominent than ${\dot{W}}_{\mathrm{tot}}$, which finally leads to the decrease in *COP*.

### 4.2. Effect of Evaporation Temperature T_evap_

The evaporation temperatures of different stages have specific impacts on the refrigeration performance, which are closely related to the compression ratios, energy cost, and refrigeration output. Concretely, *T*_evap,HTC_ controls the LTC condensation pressure through the CHX, while *T*_evap,LTC_ determines the final evaporation condition. The corresponding analyses of different stages are detailed as follows.

#### 4.2.1. HTC Evaporation Temperature *T*_evap,HTC_

**Figure 10:**
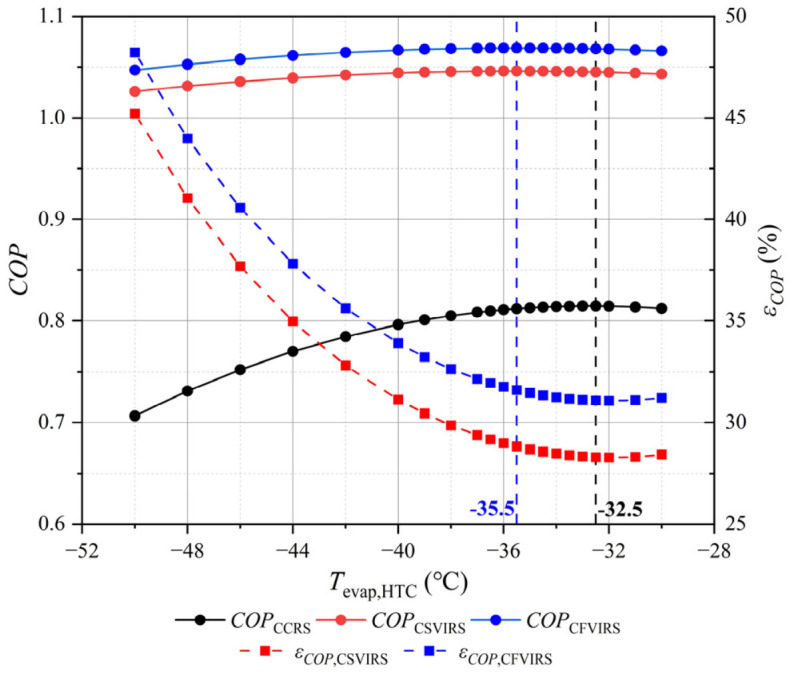
The effects of *T*_evap,HTC_ on *COP* and *ε_COP_*.

With *T*_cond_ and *T*_evap,LTC_ maintained at 30 °C and −80 °C, *T*_evap,HTC_ ranges from −50 to −30 °C, and the curves of *COP* and *ε_COP_* are depicted in Figure 10. The results indicate that as *T*_evap,HTC_ increases, the *COP* of each system initially increases and then decreases, presenting an optimal *T*_evap,HTC_. The optimal *T*_evap,HTC_ of the CSVIRS and CFVIRS are both about −35.5 °C, which are relatively lower than that of the CCRS, which is around −32.5 °C. The maximum *COP* of the CSVIRS and CFVIRS are 1.045 and 1.068, while the maximum *COP* of the CCRS is only 0.815. Additionally, the *ε_COP_* values of the CSVIRS and CFVIRS reach the minimum around the optimum *T*_evap,HTC_ of the CCRS.

**Figure 11:**
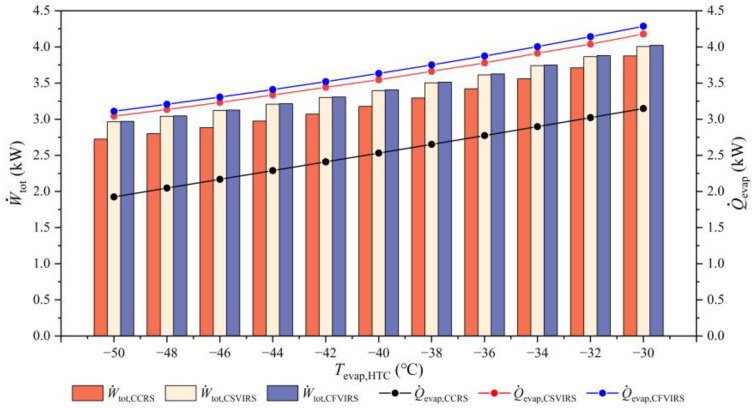
The effects of *T*_evap,HTC_ on ${\dot{W}}_{\mathrm{tot}}$ and ${\dot{Q}}_{\mathrm{evap}}$.

Figure 11 further exhibits the variations in ${\dot{W}}_{\mathrm{tot}}$ and ${\dot{Q}}_{\mathrm{evap}}$ with different *T*_evap,HTC_. Increasing *T*_evap,HTC_ alleviates the specific compression work and supplements the main flow of the HTC, which contributes to the heat transfer quantity of the CHX and enhances the main flow and refrigeration output of the LTC. Meanwhile, the compression ratio and power consumption of the LTC also increase correspondingly. Within the aforementioned range of *T*_evap,HTC_, the ${\dot{W}}_{\mathrm{tot}}$ of the CFVIRS ranges between 2.97 and 4.02 kW, which is 0.15–0.41% and 3.76–8.99% higher than the CSVIRS and CCRS, respectively. Furthermore, the refrigeration output ${\dot{Q}}_{\mathrm{evap}}$ of the CFVIRS varies from 3.11 to 4.29 kW, which exceeds that of the CSVIRS and CCRS by 2.22–2.58% and 36.15–61.53%. The growth rate of ${\dot{W}}_{\mathrm{tot}}$ is more dominant compared with ${\dot{Q}}_{\mathrm{evap}}$, which prompts the existence of optimum *T*_evap,HTC_. The ${\dot{W}}_{\mathrm{tot}}$ of the CCRS is relatively lower than that of the CSVIRS and CFVIRS mainly due to the lack of refrigerant flow rate, and this problem further restricts the evaporation capacity and exacerbates *COP* significantly.

#### 4.2.2. LTC Evaporation Temperature *T*_evap,LTC_

**Figure 12:**
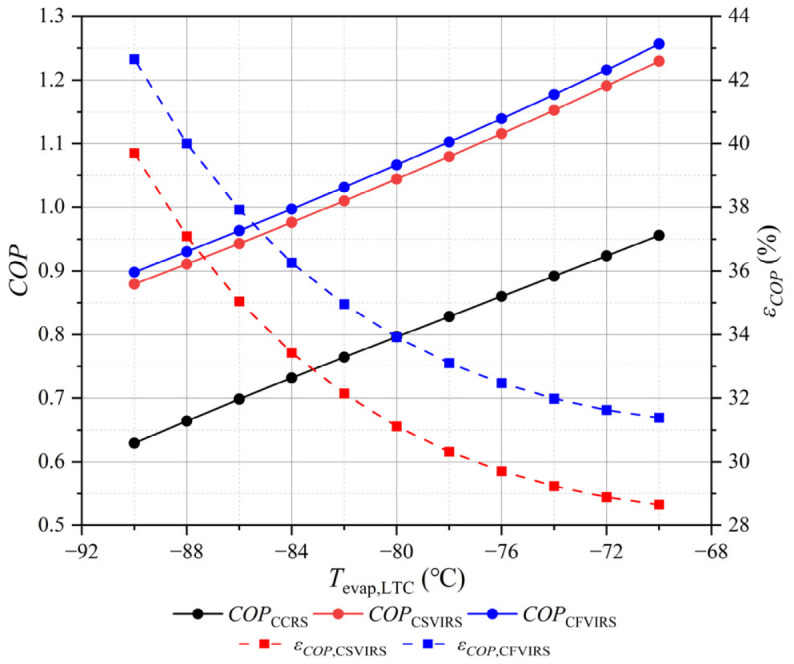
The effects of *T*_evap,LTC_ on *COP* and *ε_COP_*.

With *T*_cond_ and *T*_evap,HTC_ held at 30 and −40 °C and *T*_evap,LTC_ ranging from −90 to −70 °C, the variations in *COP* and *ε_COP_* are illustrated in Figure 12. Decreasing *T*_evap,LTC_ leads to a remarkable drop in *COP*, while the improving effects of the CSVIRS and CFVIRS demonstrate an intensified tendency at lower evaporation temperature. Under the given range of *T*_evap,LTC_, the CFVIRS exhibits *COP* improvements of 2.12–2.13% and 31.39–42.66% over the CSVIRS and CCRS, respectively.

**Figure 13:**
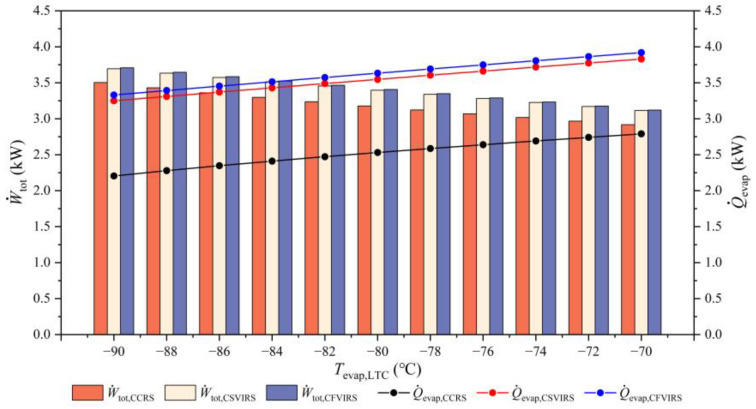
The effects of *T*_evap,LTC_ on ${\dot{W}}_{\mathrm{tot}}$ and ${\dot{Q}}_{\mathrm{evap}}$.

The effects of *T*_evap,LTC_ on ${\dot{W}}_{\mathrm{tot}}$ and ${\dot{Q}}_{\mathrm{evap}}$ are shown in Figure 13. It is indicated that ${\dot{W}}_{\mathrm{tot}}$ shows an increasing trend as *T*_evap,LTC_ decreases, owing to the rising compression ratio of the LTC. Within the given range of *T*_evap,LTC_, the ${\dot{W}}_{\mathrm{tot}}$ of the CFVIRS increases from 3.12 to 3.71 kW, which yields increment percentages of 0.19–0.37% and 5.86–6.90% over the CSVIRS and CCRS. Moreover, ${\dot{Q}}_{\mathrm{evap}}$ suffers a significant reduction mainly due to the decrease in refrigerant mass flow, which further deteriorates the refrigeration performance. The ${\dot{Q}}_{\mathrm{evap}}$ of the CFVIRS ranges between 3.33 and 3.92 kW, which is 2.33–2.50% and 40.47–51.02% higher than the CSVIRS and CCRS. Compared to the conventional system, the cascade vapor-injection configurations exhibit adequate refrigeration capacity and enhanced energy efficiency, especially under lower *T*_evap,LTC_, and the CFVIRS is recognized as the superior configuration.

### 4.3. Effect of Injection Pressure p_inj_

The intermediate injection pressures (*p*_inj_) of the HTC and LTC correspond to the injection vapor temperatures of the compressors, which can be adjusted by controlling the injection expansion valves. For the CSVIRS, while a reduction in *p*_inj_ may be beneficial for the subcooling of the evaporator, the resulting changes in refrigerant mass flow rate, entrainment ratio, and compressor workload concurrently affect the overall energy efficiency. Meanwhile, the *p*_inj_ of the CFVIRS determines the vapor quality at the inlet of the flash tank, which also controls the entrainment ratio and compression work. The initial presumption of optimal *p*_inj_ is selected as the geometric mean of the evaporation and condensation pressure in accordance with the mainstream research, but the concrete variation in performance against *p*_inj_ should be assessed. The specific evaluations are as follows.

#### 4.3.1. HTC Injection Pressure *p*_inj,HTC_

**Figure 14:**
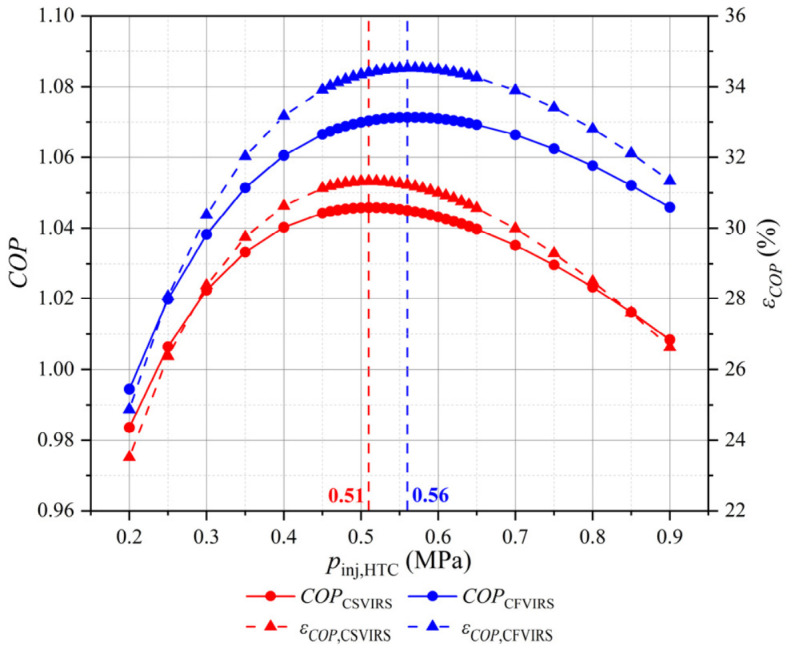
The effects of *p*_inj,HTC_ on *COP* and *ε_COP_*.

With *T*_cond_, *T*_eva,HTC_ and *T*_eva,LTC_ maintained at 30 °C/−40 °C/−80 °C, *p*_inj,HTC_ is adjusted between the evaporation and condensation pressure of the HTC. According to Figure 14, it is noticeable that the *COP* of both systems increases initially and then drops with the rise in *p*_inj,HTC_. Under the given range of *p*_inj,HTC_, the *COP* of the CSVIRS reaches the maximum value of 1.046 when *p*_inj,HTC_ = 0.51 MPa, while the optimal *COP* of the CFVIRS is comparatively higher, at 1.071, when *p*_inj,HTC_ reaches 0.56 MPa. The geometric mean of *p*_evap,HTC_ and *p*_cond,HTC_ is about 0.45 MPa, which is relatively lower than the optimal solutions of *p*_inj,HTC_ for the CSVIRS and CFVIRS. Nevertheless, the presumed optimal *COP* of the CSVIRS and CFVIRS is 0.2% and 0.5% lower than their actual optimal COP, respectively.

#### 4.3.2. LTC Injection Pressure *p*_inj,LTC_

**Figure 15:**
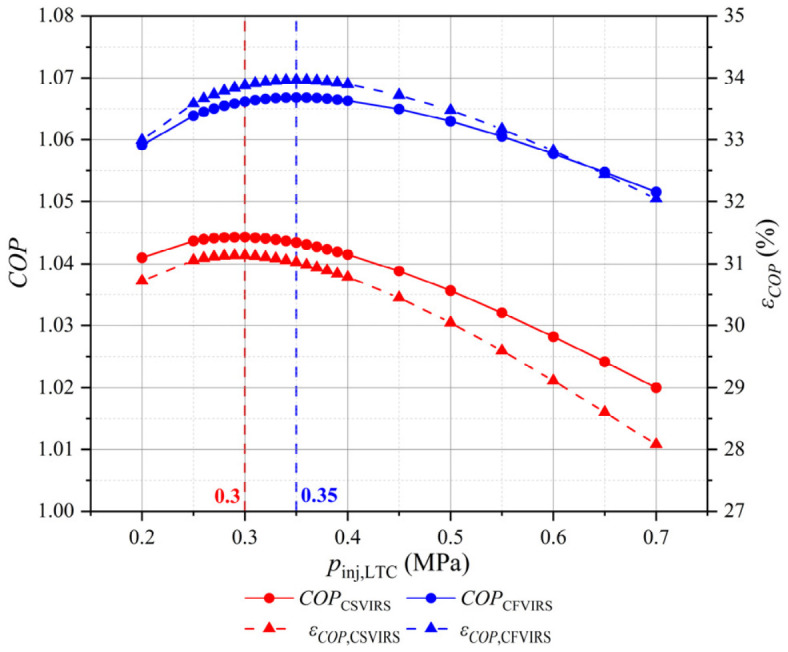
The effects of *p*_inj,LTC_ on *COP* and *ε_COP_*.

With the similar conditions set as 4.3.1, *p*_inj,LTC_ also varies between the evaporation and condensation pressure of the LTC. Represented in Figure 15, the curves of *COP* and *ε_COP_* against *p*_inj,LTC_ show similar non-monotonic trends, which are flatter than the curves of the HTC. The *COP* of the CSVIRS attains the peak value of 1.044 when *p*_inj,LTC_ = 0.3 MPa, while the optimal *COP* of the CFVIRS is slightly higher, at 1.067, when *p*_inj,LTC_ reaches 0.35 MPa. Moreover, the presumed optimal *COP* of the CSVIRS and CFVIRS exhibits merely 0.01% and 0.04% deviations below their validated optimal *COP*, respectively.

To sum up, employing the geometric mean of evaporation and condensation pressure as the default intermediate injection pressure is verified with convincing feasibility.

### 4.4. Effect of Entrainment Ratio μ

The entrainment ratio *μ* is an essential factor to control the refrigerant flow allocation, which has a substantial impact on the performance of vapor-injection refrigeration system. For the CSVIRS, the flow split condition is jointly controlled by the expansion valves of injection bypass and evaporation path. Increasing the proportion of injection flow may be profitable for the compression efficiency and subcooling effect, but the sacrifice of evaporation flow is disadvantageous to the refrigeration output. For the CFVIRS, since the input vapor quality of the flash tank is determined by *p*_inj_ because of the isenthalpic expansion procedure after condensation, the *μ*_CFVIRS_ remains stable with fixed *p*_inj_. This section mainly analyzes the effect of *μ*_CSVIRS_.

#### 4.4.1. HTC Entrainment Ratio *μ*_HTC_

**Figure 16:**
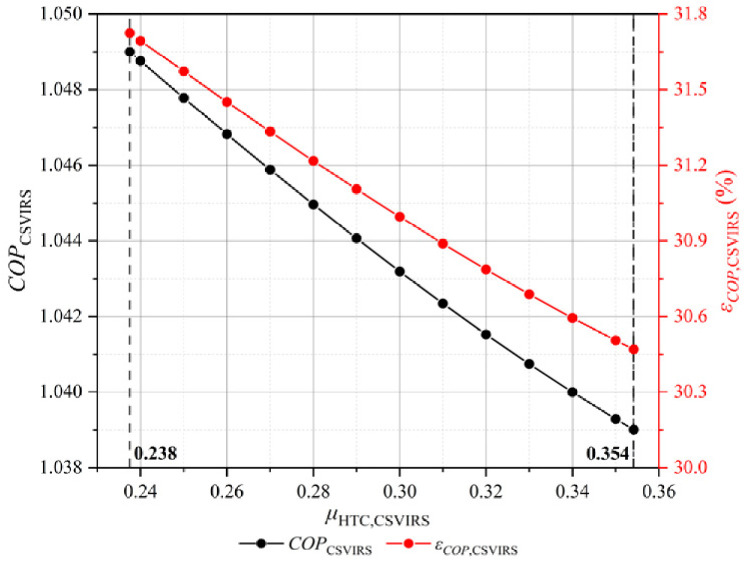
The effects of *μ*_HTC_ on *COP* and *ε_COP_* of CSVIRS.

With *T*_cond_, *T*_eva,HTC_, and *T*_eva,LTC_ kept at 30 °C/−40 °C/−80 °C, the effects of *μ*_HTC_ on the *COP* and *ε_COP_* of the CSVIRS are illustrated in Figure 16. The *COP* and *ε_COP_* curves descend as *μ*_HTC_ increases, and the minimum and maximum *COP*_CSVIRS_ are 1.039 and 1.049, which are reached at *μ*_HTC,max_ = 0.354 and *μ*_HTC,min_ = 0.238, respectively. It should be emphasized that with the assumption of ∆*T*_sub_ = 5 °C, *μ*_HTC_ encounters the minimum limit when the temperature difference between the outlet of the cold side and inlet of the hot side of subcooler 1 approaches 0 °C, which results in temperature cross-violation. Furthermore, *μ*_HTC_ reaches the maximum threshold when the starting point of the secondary compression (point 2b in Figure 2b) just transits into the two-phase state, which violates the prohibition of wet compression.

**Figure 17:**
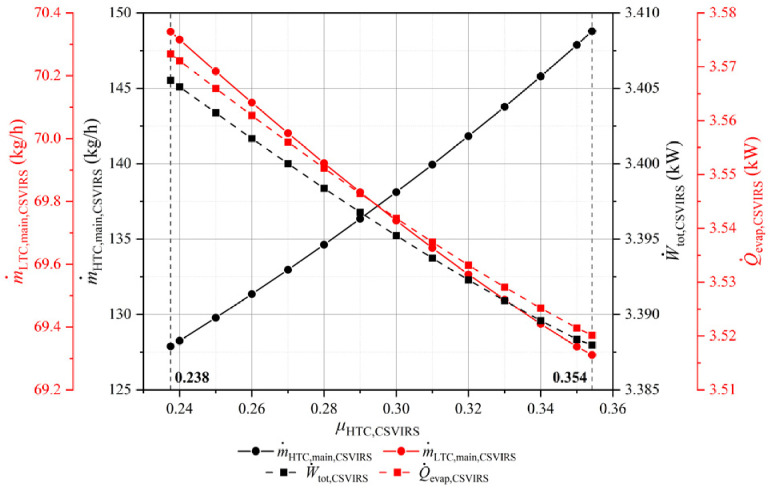
The effects of *μ*_HTC_ on ${\dot{m}}_{\mathrm{HTC},\mathrm{main}}$, ${\dot{m}}_{\mathrm{LTC},\mathrm{main}}$, ${\dot{W}}_{\mathrm{tot}}$, and ${\dot{Q}}_{\mathrm{evap}}$ of CSVIRS.

The effects of *μ*_HTC_ on the ${\dot{m}}_{\mathrm{HTC},\mathrm{main}}$, ${\dot{m}}_{\mathrm{LTC},\mathrm{main}}$, ${\dot{W}}_{\mathrm{tot}}$ and ${\dot{Q}}_{\mathrm{evap}}$ of the CSVIRS are displayed in Figure 17. As *μ*_HTC_ increases, ${\dot{m}}_{\mathrm{HTC},\mathrm{main}}$ rises from 127.89 to 148.78 kg/h, while ${\dot{m}}_{\mathrm{LTC},\mathrm{main}}$ shows a slight descent from 70.34 to 69.31 kg/h. Meanwhile, ${\dot{W}}_{\mathrm{tot}}$ decreases from 3.41 to 3.39 kW and ${\dot{Q}}_{\mathrm{evap}}$ demonstrates a sharper drop from 3.57 to 3.52 kW. It is notable that increasing *μ*_HTC_ enhances the flow rate of HTC by improving the compression efficiency, but the reduction trend of ${\dot{Q}}_{\mathrm{evap}}$ is more prominent than ${\dot{W}}_{\mathrm{tot}}$, which finally leads to the monotonic descent of *COP*_CSVIRS_.

#### 4.4.2. LTC Entrainment Ratio *μ*_LTC_

**Figure 18:**
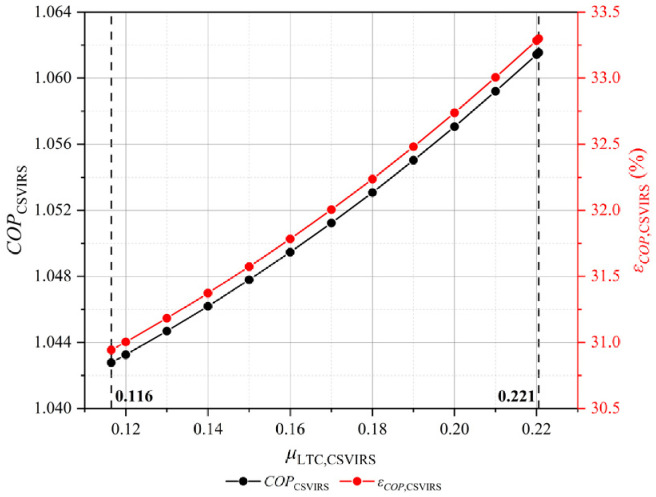
The effects of *μ*_LTC_ on *COP* and *ε_COP_* of CSVIRS.

With similar conditions as 4.4.1, the variations in *COP* and *ε_COP_* with *μ*_LTC_ of the CSVIRS are shown in Figure 18. Corresponding to the boundary verification of *μ*_HTC_, the lower and upper limits of *μ*_LTC_ are 0.116 and 0.221, respectively. The *COP* and *ε_COP_* curves ascend as *μ*_LTC_ increases, and the minimum and maximum *COP*_CSVIRS_ are 1.043 and 1.062.

**Figure 19:**
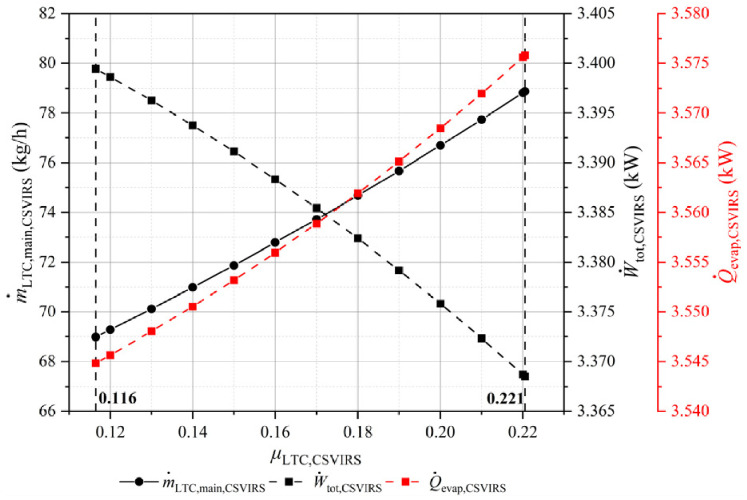
The effects of *μ*_LTC_ on ${\dot{m}}_{\mathrm{LTC},\mathrm{main}}$, ${\dot{W}}_{\mathrm{tot}}$, and ${\dot{Q}}_{\mathrm{evap}}$ of CSVIRS.

The working condition of the HTC remains stable, and the impacts of *μ*_LTC_ on ${\dot{m}}_{\mathrm{LTC},\mathrm{main}}$, ${\dot{W}}_{\mathrm{tot}}$, and ${\dot{Q}}_{\mathrm{evap}}$ of the CSVIRS are demonstrated in Figure 19. Increasing *μ*_LTC_ restricts the proportion of evaporation flow, but ${\dot{m}}_{\mathrm{LTC},\mathrm{main}}$ still rises from 68.98 to 78.88 kg/h, which finally improves ${\dot{Q}}_{\mathrm{evap}}$ from 3.54 to 3.58 kW. In addition, ${\dot{W}}_{\mathrm{tot}}$ reduces from 3.40 to 3.37 kW due to the compression enhancement of the LTC, which is also conducive to *COP*_CSVIRS_.

### 4.5. Comparative Analysis of Different Refrigerant Pairs

The aforementioned parameter discussions are initially based on the conventional combination of R143a-R23. Referring to the selected refrigerants for the HTC and LTC listed in Table 2, a total of nine different refrigerant pairs are arranged to comparatively evaluate the performance of the CFVIRS.

**Figure 20:**
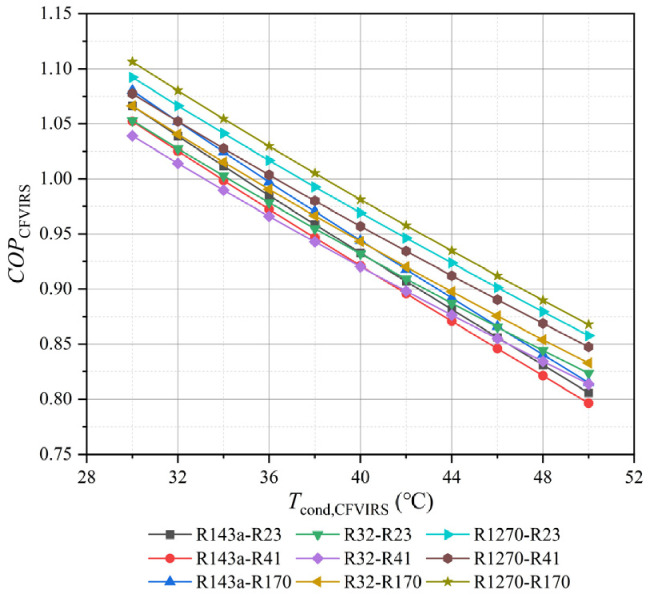
The effects of *T*_cond_ on *COP*_CFVIRS_ for different refrigerant pairs.

**Figure 21:**
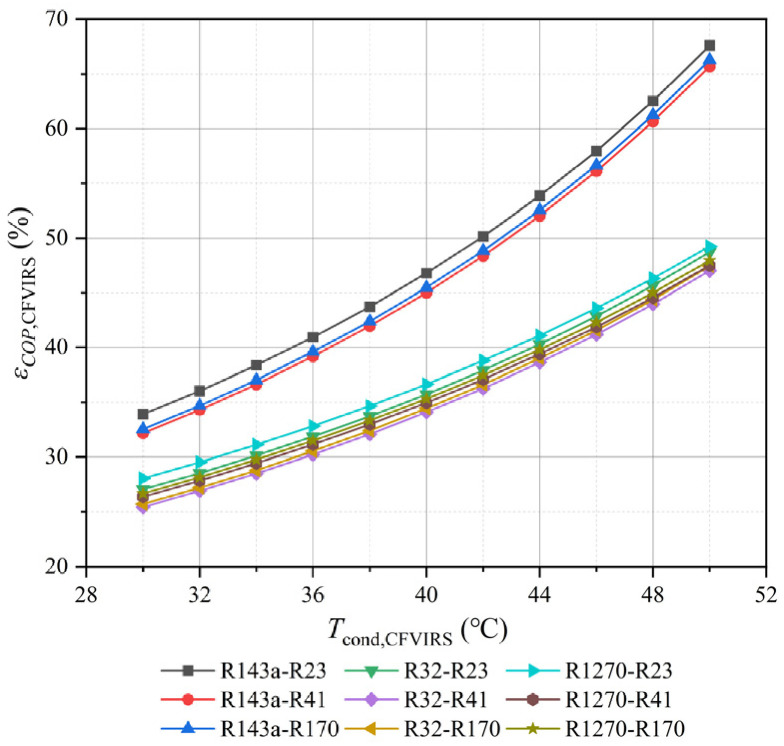
The effects of *T*_cond_ on *ε_COP_*_,CFVIRS_ for different refrigerant pairs.

The variations in *COP*_CFVIRS_ and *ε_COP_*_,CFVIRS_ versus *T*_cond_ for different refrigerant pairs are displayed in Figure 20 and Figure 21. The combination of R1270-R170 outperforms other pairs, with the highest *COP* of 1.106 when *T*_cond_ = 30 °C, while R32-R41 and R143a-R41 are identified with the poorest performance. Moreover, the optimization effect of R143a-R23 is more pronounced with the maximum *ε_COP_* of 67.60% when *T*_cond_ = 50 °C, while R32-R41 demonstrates the slightest improvement ratio.

**Figure 22:**
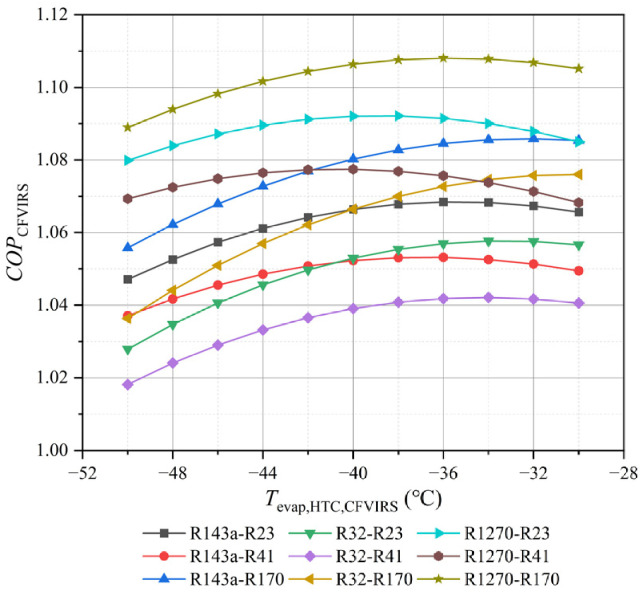
The effects of *T*_evap,HTC_ on *COP*_CFVIRS_ for different refrigerant pairs.

**Figure 23:**
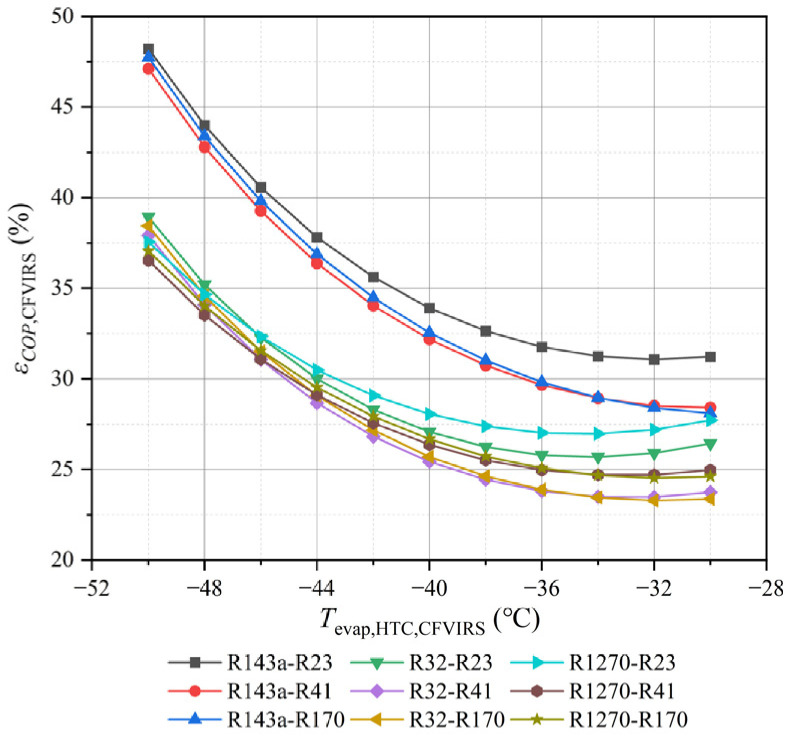
The effects of *T*_evap,HTC_ on *ε_COP_*_,CFVIRS_ for different refrigerant pairs.

The impacts of *T*_evap,HTC_ on *COP*_CFVIRS_ and *ε_COP_*_,CFVIRS_ for different refrigerant pairs are illustrated in Figure 22 and Figure 23. The *COP*_CFVIRS_ curves consistently demonstrate a similar trend of peaking and declining as 4.2.1, and the optimum *T*_evap,HTC_ for each pair differs from each other. The *COP*_CFVIRS_ of R1270-R170 exceeds other groups and attains the maximum of 1.108 with *T*_evap,HTC_ around −36 °C, which represents a 6.33% enhancement over the optimal *COP*_CFVIRS_ of R32-R41. Meanwhile, R143a-R170 and R32-R170 are verified with higher optimum *T*_evap,HTC_ among the selected pairs. The curves of *ε_COP_*_,CFVIRS_ exhibit an opposite trend compared with *COP*_CFVIRS_. Notably, all the curves demonstrate convergence characteristics in the low *T*_evap,HTC_ region, forming tightly clustered bundles. The refrigerant pairs containing R143a outperform other combinations with more pronounced improvement. The pair of R143a-R23 is observed to show the highest *ε_COP_*_,CFVIRS_ of 48.21% when *T*_evap,HTC_ = −50 °C.

**Figure 24:**
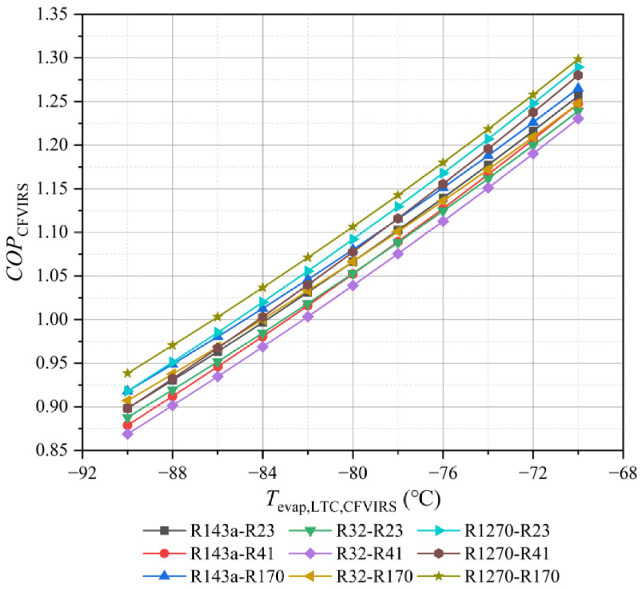
The effects of *T*_evap,LTC_ on *COP*_CFVIRS_ for different refrigerant pairs.

**Figure 25:**
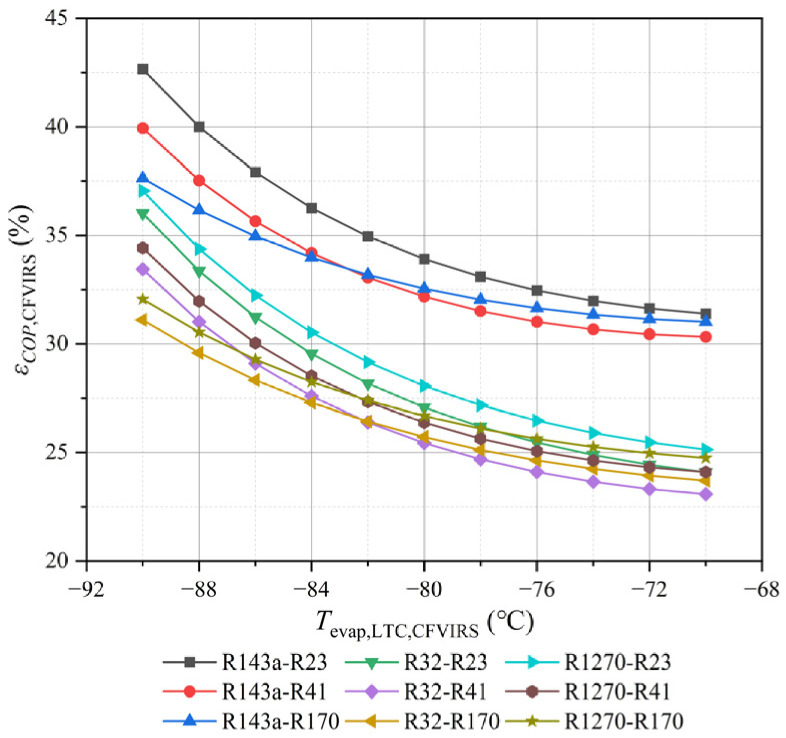
The effects of *T*_evap,LTC_ on *ε_COP_*_,CFVIRS_ for different refrigerant pairs.

The curves of *COP*_CFVIRS_ and *ε_COP_*_,CFVIRS_ versus *T*_evap,LTC_ for different refrigerant pairs are plotted as Figure 24 and Figure 25. Since the decrease in *T*_evap,LTC_ leads to a reduction in *COP*_CFVIRS_, the maximum *COP*_CFVIRS_ of 1.299 is attained at *T*_evap,LTC_ = −70 °C for R1270-R170. The curves of *ε_COP_*_,CFVIRS_ exhibit similar convergence features in the high *T*_evap,LTC_ region, and R143a-R23 achieves the highest *ε_COP_*_,CFVIRS_ of 42.66% at *T*_evap,LTC_ = −90 °C.

### 4.6. Exergy Analysis

Established in the parameter evaluations referring to the first law of thermodynamics, in-depth discussions are urged on the exergy losses incurred by different components of the systems. This analysis aims to clarify the reversibility of the refrigeration systems and evaluate the exergy degradation, providing advanced insights for optimizing energy efficiency, sustainability, and operational cost-effectiveness.

**Figure 26:**
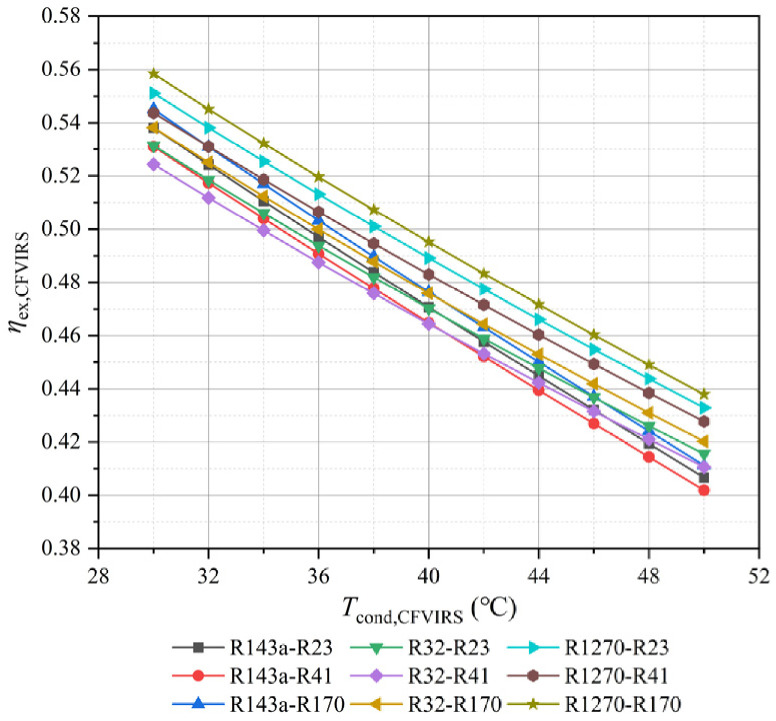
The effects of *T*_cond_ on *η*_ex,CFVIRS_ for different refrigerant pairs.

**Figure 27:**
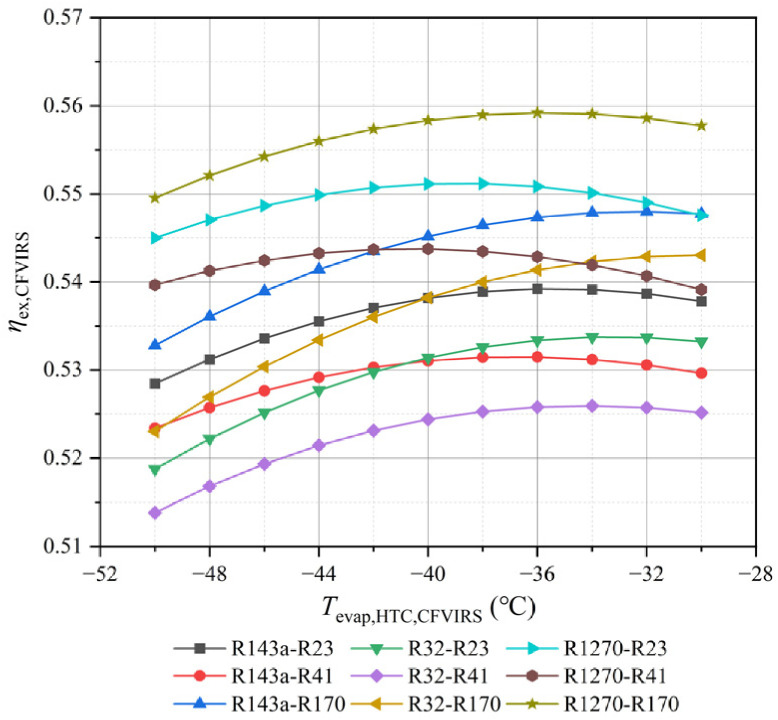
The effects of *T*_evap,HTC_ on *η*_ex,CFVIRS_ for different refrigerant pairs.

**Figure 28:**
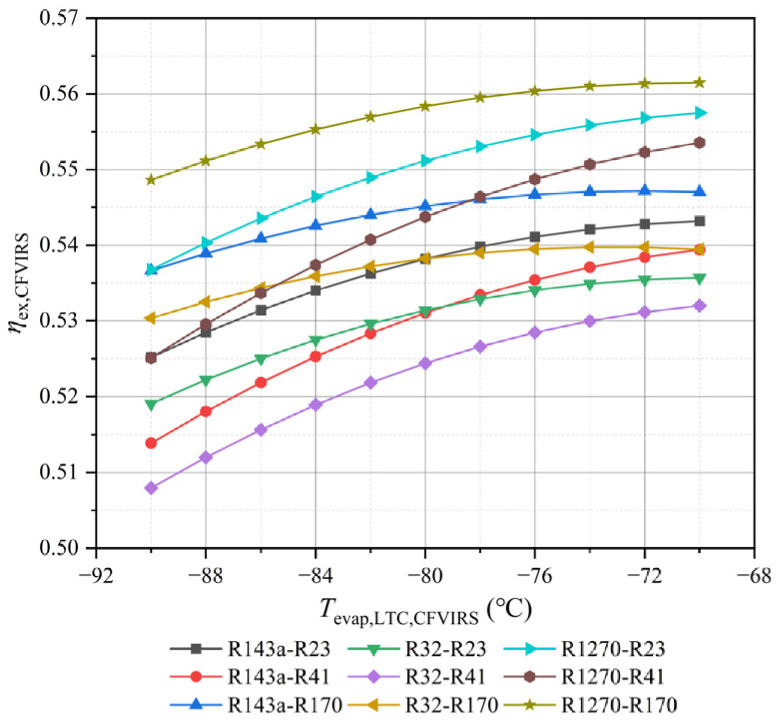
The effects of *T*_evap,LTC_ on *η*_ex,CFVIRS_ for different refrigerant pairs.

Based on the configuration of the CFVIRS, the effects of *T*_cond_, *T*_evap,HTC_, and *T*_evap,LTC_ on *η*_ex,CFVIRS_ for different refrigerant pairs are presented in Figure 26, Figure 27 and Figure 28. It is notable that *η*_ex,CFVIRS_ descends with the rise in *T*_cond_, and the curve of R1270-R170 still exceeds other combinations, with the maximum *η*_ex,CFVIRS_ of 0.558 attained at *T*_cond_ = 30 °C. Moreover, the variation in *η*_ex,CFVIRS_ exhibits a corresponding trend of rising and falling as *COP* versus *T*_evap,HTC_, and the optimal *η*_ex,CFVIRS_ of R1270-R170 is 0.559 with *T*_evap,HTC_ around −36 °C. Since the changes in *T*_cond_ and *T*_evap,HTC_ are irrelevant to the refrigeration temperature, *η*_ex,CFVIRS_ is dominantly controlled by *COP* to reflect the transformation ratio between the electric power input and cold exergy output. Nevertheless, the ascending curves of *η*_ex,CFVIRS_ become flatter as *T*_evap,LTC_ increases, and the maximum *η*_ex,CFVIRS_ of R1270-R170 is about 0.562 while *T*_evap,LTC_ reaches −70 °C. More specifically, the *η*_ex,CFVIRS_ of R143a-R170 and R32-R170 even peak and then drop within the discussion range of *T*_evap,LTC_. Although increasing *T*_evap,LTC_ seems beneficial for *COP*, the cold exergy suffers a significant decline due to the narrowing gap between the refrigeration temperature and reference temperature, which reduces the overall exergy efficiency of the system.

**Figure 29:**
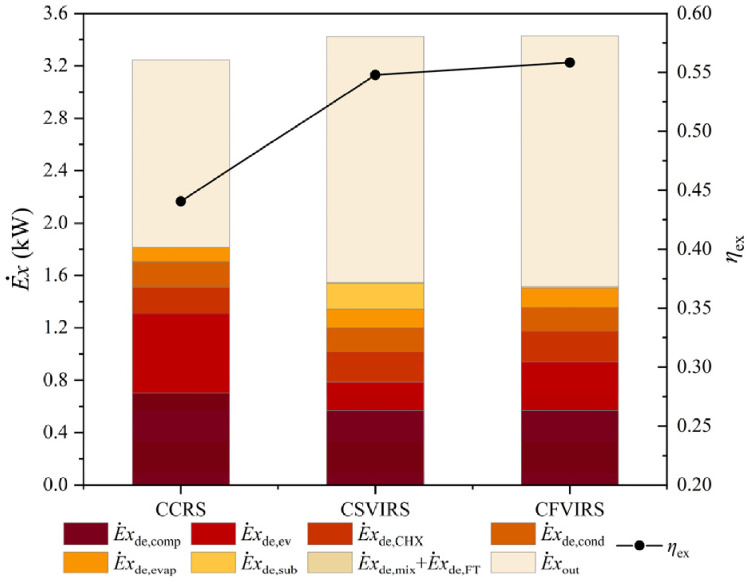
Exergy compositions and *η*_ex_ comparisons of different systems using R1270-R170.

Based on the combination of R1270-R170 under the default condition, the exergy composition and *η*_ex_ comparison of different cascade refrigeration systems are depicted in Figure 29. The summation of exergy streams $\sum \dot{E}x$ equals that of ${\dot{W}}_{\mathrm{tot}}$, and the CFVIRS is validated with the highest exergy consumption of 3.43 kW. The compressors occupy the largest percentage of exergy destruction, and the $\dot{E}x_{\mathrm{de},\mathrm{comp}}$ of the CCRS, CSVIRS, and CFVIRS are about 0.71 kW, 0.57 kW and 0.57 kW, respectively. The expansion valves account for an essential proportion of exergy destruction owing to the energy loss of isenthalpic expansion. In particular, the $\dot{E}x_{\mathrm{de},\mathrm{ev}}$ of the CSVIRS is notably less than other systems, which is only 0.22 kW compared with $\dot{E}x_{\mathrm{de},\mathrm{ev},\mathrm{CCRS}}=0.61\;\mathrm{kW}$ and $\dot{E}x_{\mathrm{de},\mathrm{ev},\mathrm{CFVIRS}}=0.37\;\mathrm{kW}$. The heat exchangers consist of condenser, evaporator, CHX, and subcoolers, which also comprise a considerable part of exergy destruction due to the heat transfer loss. The CSVIRS is verified with the highest $\dot{E}x_{\mathrm{de}}$ from heat exchangers, of about 0.75 kW, which is mainly attributed to the loss of subcoolers. The exergy output of the CFVIRS is 1.91 kW, while the exergy output of the CCRS and CSVIRS is 1.43 kW and 1.88 kW. In addition, the *η*_ex_ of the CFVIRS is about 0.558, which exceeds that of the CCRS and CSVIRS by 2.10% and 33.84%. Collectively, the cascade vapor-injection approach substantially reduces exergy destruction in compressors and expansion valves, and the exergy utilization effectiveness is notably strengthened.

**Figure 30:**
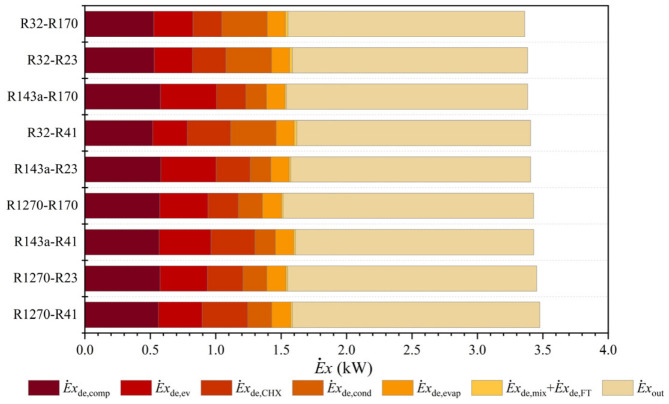
Exergy composition comparison of CFVIRS for different refrigerant pairs.

The exergy composition comparisons of the CFVIRS for different refrigerant pairs are presented in Figure 30. The exergy destruction in mixers and flash tanks is negligible and is thus grouped in the analysis. R1270-R41 and R32-R170 are observed to demonstrate the highest and lowest total exergy consumption, about 3.48 kW and 3.36 kW. R32-R41 is acknowledged to demonstrate the highest exergy destruction, about 1.62 kW, while R1270-R170 is distinguished with the lowest exergy destruction of 1.51 kW. Moreover, R1270-R170 exceeds other selections with the highest exergy output of 1.91 kW, and R32-R41 is identified with the lowest exergy output of 1.79 kW. Regarding the specific sources of exergy losses, the compressor accounts for the largest proportion among all components, while the expansion valves contribute the second largest share of exergy destruction. The pressure variations during compression and expansion are the primary causes of exergy deterioration. Moreover, the exergy waste associated with heat transfer losses in the aforementioned heat exchangers is also significant and requires targeted optimization measures. Additionally, the refrigerant pairs containing R143a are quantified with higher exergy destruction of compressor and expansion valves. Although the refrigerant pairs containing R32 are observed to demonstrate lower exergy destruction of compressor and expansion valves, the exergy destruction of heat exchangers is obviously higher than other groups.

In summary, the exergy assessment of cascade vapor-injection configurations under diverse operating conditions provides a robust basis for enhancing the comprehensive energy performance. This optimization process simultaneously reinforces cost-effectiveness and advances the environmental sustainability of contemporary cascade refrigeration systems.

## 5. Conclusions

This paper presents a systematic thermodynamic investigation of enhanced two-stage cascade refrigeration systems incorporating vapor-injection technology for low-temperature applications. Two cascade vapor-injection structures equipped with subcoolers and flash tanks (CSVIRS and CFVIRS) are comprehensively investigated and compared with the conventional cascade refrigeration system (CCRS). The simulation models incorporating energy and exergy analyses are established in Aspen HYSYS to evaluate the thermodynamic performance of different refrigeration systems. The optimum operating parameters for performance improvement are identified through a comprehensive evaluation of key influencing factors and refrigerant combinations. The conclusions are summarized as follows:

- Under identical operating conditions for R143a-R23, the CSVIRS and CFVIRS are proven to demonstrate feasible enhancements compared with the CCRS, and the CFVIRS is verified to demonstrate the best performance. When *T*_cond_, *T*_eva,HTC_, and *T*_eva,LTC_ are set as 30 °C/−40 °C/−80 °C, The *COP* of the CFVIRS is 1.066, surpassing that of the CSVIRS and CCRS by 2.13% and 33.92%. The improving effect of vapor-injection configuration is more prominent when operating under higher *T*_cond_ and lower *T*_eva,LTC_, while *T*_eva,HTC_ is validated with an optimum solution.
- The effects of key parameters, including injection pressures and entrainment ratios, are extensively evaluated. The optimum injection pressures of the CSVIRS and CFVIRS are relatively higher than the geometric mean of the evaporation and condensation pressures. For the CSVIRS, decreasing the entrainment ratio of the HTC and increasing the entrainment ratio of the LTC within the appropriate ranges are beneficial for *COP*.
- The performance of the CFVIRS is comparatively evaluated based on various combinations of refrigerants under different condensation and evaporation temperatures. R1270-R170 exhibits the highest *COP* among the tested groups, attaining the maximum value of 1.299 with *T*_evap,LTC_ = −70 °C. Meanwhile, R143a-R23 demonstrates the highest improvement ratio of *COP*.
- Through exergy analysis, the effects of *T*_cond_, *T*_evap,HTC_, and *T*_evap,LTC_ on the exergy efficiency *η*_ex_ of the CFVIRS with different refrigerant pairs are comparatively discussed. *T*_cond_ and *T*_evap,HTC_ primarily influence *η*_ex_ by altering *COP*, while *T*_evap,LTC_ also controls the output cold exergy. The *η*_ex_ of R1270-R170 attains the maximum value of 0.562 while *T*_evap,LTC_ = −70 °C.
- The exergy flows and destruction compositions of different refrigeration systems are analyzed in detail. The cascade vapor-injection strategy effectively alleviates exergy destruction in compressors and expansion valves. For R1270-R170, the *η*_ex_ of the CFVIRS surpasses that of the CCRS and CSVIRS by 33.84% and 2.10% under default conditions.
- The exergy composition comparisons of the CFVIRS for different refrigerant pairs are also examined. The combinations of R1270-R41 and R32-R170 are observed to demonstrate the highest and lowest total exergy consumption, while R32-R41 and R1270-R170 are observed to demonstrate the highest and lowest exergy destruction. Moreover, R1270-R170 and R32-R41 are confirmed to have the highest and lowest exergy output.

In addition, due to space limitations and the need for computational efficiency, certain conditions have been idealized and simplified using empirical formulas and theoretical models. Future studies should combine experimental validation with theoretical analysis to verify the model’s feasibility, thereby promoting the broader implementation of vapor-injection technology in refrigeration applications.

## Figures and Tables

**Table 1:** The exergy balance equations and destruction analyses of each component.

Components	Exergy Destruction
Compressor	$\dot{E}x_{\mathrm{de},\mathrm{comp}}=({\dot{m}}_{\mathrm{comp},\mathrm{out}}s_{\mathrm{comp},\mathrm{out}}-\sum {\dot{m}}_{\mathrm{comp},\mathrm{in}}s_{\mathrm{comp},\mathrm{in}})T_{0}$
Condenser	$\dot{E}x_{\mathrm{de},\mathrm{cond}}={\dot{m}}_{\mathrm{cond}}[(h_{\mathrm{cond},\mathrm{in}}-h_{\mathrm{cond},\mathrm{out}})-T_{0}(s_{\mathrm{cond},\mathrm{in}}-s_{\mathrm{cond},\mathrm{out}})]$
CHX/subcooler	$\begin{array}{ll} \dot{E}x_{\mathrm{de},\mathrm{CHX}/\mathrm{sub}} & ={\dot{m}}_{\mathrm{CHX}/\mathrm{sub},\mathrm{cold}}[(h_{\mathrm{CHX}/\mathrm{sub},\mathrm{cold},\mathrm{in}}-h_{\mathrm{CHX}/\mathrm{sub},\mathrm{cold},\mathrm{out}})-T_{0}(s_{\mathrm{CHX}/\mathrm{sub},\mathrm{cold},\mathrm{in}}-s_{\mathrm{CHX}/\mathrm{sub},\mathrm{cold},\mathrm{out}})]\\ & \;+{\dot{m}}_{\mathrm{CHX}/\mathrm{sub},\mathrm{hot}}[(h_{\mathrm{CHX}/\mathrm{sub},\mathrm{hot},\mathrm{in}}-h_{\mathrm{CHX}/\mathrm{sub},\mathrm{hot},\mathrm{out}})-T_{0}(s_{\mathrm{CHX}/\mathrm{sub},\mathrm{hot},\mathrm{in}}-s_{\mathrm{CHX}/\mathrm{sub},\mathrm{hot},\mathrm{out}})] \end{array}$
Expansion valve	$\dot{E}x_{\mathrm{de},\mathrm{EV}}={\dot{m}}_{\mathrm{EV}}T_{0}(s_{\mathrm{EV},\mathrm{out}}-s_{\mathrm{EV},\mathrm{in}})$
Evaporator	$\dot{E}x_{\mathrm{de},\mathrm{evap}}={\dot{m}}_{\mathrm{evap}}[(h_{\mathrm{evap},\mathrm{in}}-h_{\mathrm{evap},\mathrm{out}})-T_{0}(s_{\mathrm{evap},\mathrm{in}}-s_{\mathrm{evap},\mathrm{out}})]+{\dot{Q}}_{\mathrm{evap}}(1-\frac{T_{0}}{T_{\mathrm{evap}}+\Delta T_{\mathrm{evap}}})$
Flash tank	$\begin{array}{ll} \dot{E}x_{\mathrm{de},\mathrm{FT}} & ={\dot{m}}_{\mathrm{FT},\mathrm{in}}(h_{\mathrm{FT},\mathrm{in}}-T_{0}s_{\mathrm{FT},\mathrm{in}})-[{\dot{m}}_{\mathrm{FT},\mathrm{gas},\mathrm{out}}(h_{\mathrm{FT},\mathrm{gas},\mathrm{out}}-T_{0}s_{\mathrm{FT},\mathrm{gas},\mathrm{out}})\\ & \;+{\dot{m}}_{\mathrm{FT},\mathrm{liq},\mathrm{out}}(h_{\mathrm{FT},\mathrm{liq},\mathrm{out}}-T_{0}s_{\mathrm{FT},\mathrm{liq},\mathrm{out}})] \end{array}$
Mixer	$\dot{E}x_{\mathrm{de},\mathrm{mix}}=\sum {\dot{m}}_{\mathrm{mix},\mathrm{in}}(h_{\mathrm{mix},\mathrm{in}}-T_{0}s_{\mathrm{mix},\mathrm{in}})-{\dot{m}}_{\mathrm{mix},\mathrm{out}}(h_{\mathrm{mix},\mathrm{out}}-T_{0}s_{\mathrm{mix},\mathrm{out}})$
Total exergy destruction	$\begin{array}{ll} \dot{E}x_{\mathrm{de},\mathrm{tot}} & =\sum \dot{E}x_{\mathrm{de},\mathrm{comp}}+\dot{E}x_{\mathrm{de},\mathrm{cond}}+\sum \dot{E}x_{\mathrm{de},\mathrm{evap}}+\sum \dot{E}x_{\mathrm{de},\mathrm{EV}}+\sum \dot{E}x_{\mathrm{de},\mathrm{sub}}+\dot{E}x_{\mathrm{de},\mathrm{CHX}}\\ & \;\;\;\;+\sum \dot{E}x_{\mathrm{de},\mathrm{FT}}+\sum \dot{E}x_{\mathrm{de},\mathrm{mix}} \end{array}$

**Table 2:** The refrigerant parameters of HTC and LTC [35].

Refrigerants		Type	Molar Mass (kg⋅kmol^−1^)	NBP (°C)	*T*_crit_ (°C)	*p*_crit_ (MPa)	Normal Latent Heat (kJ⋅kg^−1^)	ASHRAE Safety Group	ODP	GWP
HTC	R143a	HFC	84	−47.2	72.7	3.76	226.7	A2L	0	4470
	R1270	HC	42	−47.6	91.1	4.56	438.9	A3	0	1.8
	R32	HFC	52	−51.7	78.1	5.78	381.9	A2L	0	675
LTC	R41	HFC	34	−78.3	44.1	5.91	487.8	A3	0	92
	R23	HFC	70	−82.0	26.1	4.83	239.4	A1	0	14,800
	R170	HC	30	−88.6	32.2	4.87	489.4	A3	0	5.5

**Table 3:** *COP* comparison of simulation data with reference [65].

*T*_cond_ (°C)	Reference Paper	Present Work	Relative Error (%)
20	1.366	1.352	−1.02
22	1.320	1.307	−1.00
24	1.278	1.265	−1.00
26	1.236	1.224	−0.98
28	1.197	1.184	−1.04
30	1.159	1.146	−1.06
32	1.122	1.110	−1.04
34	1.087	1.075	−1.16
36	1.051	1.040	−1.04
38	1.018	1.007	−1.05
40	0.986	0.975	−1.06

## Data Availability

Data will be made available on request.

## Nomenclature

h	specific enthalpy (kJ⋅kg^−1^)
$\dot{m}$	mass flow rate (kg⋅s^−1^)
p	pressure (MPa)
$\dot{Q}$	heat transfer rate (kW)
r	pressure ratio
s	specific entropy (kJ⋅kg^−1^⋅K^−1^)
T	temperature (°C)
u	velocity (m⋅s^−1^)
$\dot{W}$	power (kW)
x	vapor quality
Δ	difference
ex	specific exergy (kJ⋅kg^−1^)
$\dot{E}x$	exergy rate (kW)
COP	coefficient of performance
Greek symbols	
η	efficiency
μ	entrainment ratio
δ	percentage
ε	effectiveness
Subscripts	
0	reference state
a, b, 1, 2, 3…	state points
de	destruction
ex	exergy
in	inlet
is	isentropic process
gas	gas phase
hot	hot side
inj	injection bypass
liq	liquid phase
max	maximum value
min	minimum value
net	net input/output
out	outlet
set	preset condition
tot	total
cold	cold side
comp	compressor
cond	condenser
crit	critical point
evap	evaporator
main	main stream
Abbreviations	
EV	expansion valve
FT	flash tank
CHX	cascade heat exchanger
GWP	global warming potential
HTC	high-temperature cycle
LTC	low-temperature cycle
NBP	normal boiling point
ODP	ozone depletion potential
CCRS	conventional cascade refrigeration system
CFVIRS	cascade flash tank vapor-injection refrigeration system
CSVIRS	cascade subcooler vapor-injection refrigeration system
